# Objective Bayesian Edge Screening and Structure Selection for Ising Networks

**DOI:** 10.1007/s11336-022-09848-8

**Published:** 2022-02-22

**Authors:** M. Marsman, K. Huth, L. J. Waldorp, I. Ntzoufras

**Affiliations:** 1 University of Amsterdam, Psychological Methods, Nieuwe Achtergracht 129B, PO Box 15906, 1001 NK Amsterdam, The Netherlands; 2 Centre for Urban Mental Health, Amsterdam, The Netherlands; 3 Athens University of Economics and Business, Athens, Greece

**Keywords:** Bayesian model selection, ising model, spike and slab prior, depression, alcohol use disorder

## Abstract

The Ising model is one of the most widely analyzed graphical models in network psychometrics. However, popular approaches to parameter estimation and structure selection for the Ising model cannot naturally express uncertainty about the estimated parameters or selected structures. To address this issue, this paper offers an objective Bayesian approach to parameter estimation and structure selection for the Ising model. Our methods build on a continuous spike-and-slab approach. We show that our methods consistently select the correct structure and provide a new objective method to set the spike-and-slab hyperparameters. To circumvent the exploration of the complete structure space, which is too large in practical situations, we propose a novel approach that first screens for promising edges and then only explore the space instantiated by these edges. We apply our proposed methods to estimate the network of depression and alcohol use disorder symptoms from symptom scores of over 26,000 subjects.

**Supplementary Information:**

The online version contains supplementary material available at 10.1007/s11336-022-09848-8.

Undirected graphical models, also known as Markov random fields (MRFs; Kindermann & Snell, 1980), have become an indispensable tool to describe the complex interplay of variables in many fields of science. The Ising model (Ising , 1925), or quadratic exponential model (Cox, 1972), is one MRF that attracted the interest of psychologists. It is defined by the following probability distribution over the configurations of a *p*-dimensional vector $\mathbf {x}$, with $\mathbf {x} \in \{0\text {, }1\}^p$,

1 $$\begin{aligned} p(\mathbf {x} \mid \varvec{\mu }\text {, }\varvec{\Sigma }) =\frac{1}{\text {Z}(\varvec{\mu }\text {, }\varvec{\Sigma })}\, \exp \left( \sum _{i=1}^px_i\mu _i + \sum _{i=1}^{p-1}\sum _{j=i+1}^px_ix_j\sigma _{ij}\right) , \end{aligned}$$

which covers all main effects $\mu _i$ and pairwise associations $\sigma _{ij}$ of the *p* binary variables. The pairwise associations encode the conditional dependence and independence relations between variables in the model: If an association is equal to zero, the two variables are independent given the rest of the variables, and there is no direct relation between them. Otherwise, the two variables are directly related. These relations can be visualized as edges in a network, where the model’s variables populate the network’s nodes. This view of the Ising model in psychological applications inspired the field of network psychometrics (Epskamp, Maris, Waldorp, & Borsboom, 2018; Marsman et al., 2015), which now spans research in, among others, personality (Constantini et al., 2012; Cramer et al., 2019), psychopathology (Borsboom & Cramer, 2013; Cramer et al.,^2016^), attitudes (Dalege et al., 2019; Dalege, Borsboom, van Harreveld, & van der Maas, 2016), educational measurement (Marsman, Maris, Bechger, & Glas, 2015; Marsman, Tanis, Bechger, & Waldorp, 2019), and intelligence (Savi, Marsman, van der Maas, & Maris, 2019; van der Maas, Kan, Marsman, & Stevenson, 2017).

The primary objective in empirical applications of the Ising model is determining the network’s structure or topology. Three practical challenges complicate this objective. The first practical challenge is the normalizing constant $\text {Z}(\varvec{\mu }\text {, }\varvec{\Sigma })$ in Eq. (1), which is a sum over all $2^p$ possible configurations of the binary vector $\mathbf {x}$. Even for small graphs, this normalizing constant can be expensive to compute. For example, for a network of 20 variables, the normalizing constant consists of more than one million terms. Given that the normalizing constant is repeatedly evaluated in numerical optimization or simulation approaches to estimate the model’s parameters, the direct computation of the likelihood is computationally intractable. The second practical challenge in determining the Ising model’s structure is the balance between model complexity and data. With *p* main effects and ${p\atopwithdelims ()2}$ pairwise interactions, the number of free parameters can quickly overwhelm the limited information in available data. The third practical challenge is the efficient selection of a structure with desirable statistical properties from the vast space of possible structures. For a network of 20 variables, the structure space comprises $2^{190} = 1.57 \times 10^{57}$ potential structures, which is simply too large to enumerate in practice.

In psychology, eLasso (van Borkulo et al., 2014) is the structure selection solution for the Ising model and overcomes all three challenges. First, it adopts a pseudolikelihood approach to circumvent the normalizing constant. The pseudolikelihood replaces the joint distribution of the vector variable $\mathbf {x}$—i.e., the full Ising model in Eq. (1)—with its respective full-conditional distributions:

2 $$\begin{aligned} p^*(\mathbf {x}&\mid \varvec{\mu }\text {, }\varvec{\Sigma }) \propto \prod _{i=1}^p p(x_i \mid \mathbf {x}^{(i)}\text {, }\mu _i\text {, }\varvec{\sigma }_i^{(i)}) = \frac{\exp \left( \sum _{i=1}^px_i\mu _i + \sum _{i=1}^{p-1}\sum _{j=i+1}^p x_ix_j\sigma _{ij} \right) }{\prod _{i=1}^p\left( 1+\exp \left( \mu _i + \sum _{j \ne i}\sigma _{ij}x_j\right) \right) }, \end{aligned}$$

where $\varvec{\sigma }_i^{(i)} = (\sigma _{i1}\text {, }\dots \text {, }\sigma _{i(i-1)}\text {, }\sigma _{i(i+1)}\text {, }\dots \sigma _{ip})^\mathsf{T}$. Observe that the pseudolikelihood is equivalent to Eq. (1) except that it replaces the intractable normalizing constant with a tractable one. Second, eLasso balances structure complexity with the information available from the data at hand using the Lasso (Tibshirani, 1996): An $l_1$-penalty is stipulated on the pseudolikelihood parameters (i.e., minimize $-\ln p^*(x_i \mid \mu _i\text {, }\varvec{\sigma }_i^{(i)})$ subject to the constraint $\sum _{j \ne i} |\sigma _{ij}| \le \rho$) to effectively shrink negligible effects to precisely zero. Ravikumar, Wainwright, and Lafferty (2010) showed that the pseudolikelihood in combination with Lasso can consistently uncover the true topology (see also Meinshausen & Bühlmann, 2006). Third, eLasso selects the structure that optimizes the parameters subject to the $l_1$ constraint, which is specified up to its tuning parameter $\rho$. It performs the optimization for a range of values for the tuning parameter and then selects the value that minimizes an extended Bayesian information criterion (Barber & Drton, 2015; Chen & Chen, 2008). Thus, structure selection with eLasso is analogous to selecting the tuning parameter. This combination of methods allows eLasso to efficiently perform structure selection for the Ising model, which is why it has become widely popular in psychometric practice.

We, however, have two concerns with frequentist regularization methods for estimating the Ising model, such as those used by eLasso. Our first concern is that traditional, frequentist approaches cannot express the uncertainty associated with a selected structure, and thus do not inform us about other structures that might be plausible for the data at hand. A structure’s plausibility is disclosed in its posterior probability. To compute posterior probabilities, we have to entertain multiple structures and take their prior plausibility into account. But eLasso searches for a single optimal structure instead. Our second concern is that eLasso does not articulate the precision of the parameters it estimates. Standard expressions for parameter uncertainty are unavailable for Lasso estimation (Tibshirani, 1996), since the limiting distribution of the Lasso estimator is non-Gaussian with a point mass at zero (e.g., Knight & Fu, 2000; Pötscher & Leeb, 2009). Basic solutions such as the bootstrap, although frequently used (see, for instance, Epskamp, Borsboom, & Fried,^2018^; Tibshirani, 1996), can therefore not be used to obtain confidence intervals or standard errors (e.g., Bühlmann, Kalisch, and Meier, 2014, Section 3.1; Pötscher and Leeb, 2009, Williams, 2021). Bayesian formulations of the Lasso offer a more natural framework for uncertainty quantification (Kyung, Gill, Ghosh, & Casella, 2010; Park & Casella, 2008; van Erp, Oberski, & Mulder, 2019), but approximate confidence intervals/standard errors could also be obtained by desparsifying the Lasso (Bühlmann et al., 2014; van de Geer, Bülmann, Ritov, & Dezeure, 2014).

In light of these concerns, our goals are threefold. Our primary goal is to introduce a new Bayesian approach for learning the topology of Ising models. Bayesian approaches to model selection often introduce binary indicators $\gamma$ for the selection of variables in the model (e.g., George & McCulloch, 1993; O‘Hara & Sillanpää, 2009). We will use these indicators here to model edge selection: If the indicator $\gamma _{ij}$ equals one, the edge between variables *i* and *j* is included. Otherwise, the edge is excluded. A structure *s* is then a specific configuration of a vector of ${p\atopwithdelims ()2}$ indicator variables $\varvec{\gamma }_s$, and the collection of network structures is equal to

$$\mathcal {S} = \{0\text {, }1\}^{{p\atopwithdelims ()2}}.$$

We wish to estimate the posterior structure probabilities $p(\varvec{\gamma } \mid \mathbf {x})$, since they convey all the information that is available on the structures $\varvec{\gamma } \in \mathcal {S}$ and can be used to express the plausibility of a particular structure or the inclusion of a specific edge for the data at hand. To unlock these Bayesian benefits (see Marsman & Wagenmakers, 2017; Wagenmakers, Marsman, et al., 2018, for detailed examples), we have to connect the indicator variables to the selection problem at hand.

Our secondary goal is to formulate a continuous spike-and-slab approach, initially proposed by George and McCulloch (1993) for covariate selection in regression models, for edge selection in Ising networks. In this approach, the binary indicators are used to hierarchically model the prior distributions of focal parameters by assigning zero-centered diffuse priors to effects that should be included and priors that are sharply peaked about zero to negligible effects. These continuous spike-and-slab components are usually Gaussian (e.g., George & McCulloch, 1993; Ročková & George, 2014) or Laplace distributions (e.g., Ročková, 2018; Ročková & George, 2018). Even though the Laplace distribution generates a Bayesian Lasso (Park & Casella, 2008), its drawback is that its posterior distribution is difficult to approximate using computational tools other than simulation. We therefore adopt Gaussian spike-and-slab components in our edge selection approach.

Our tertiary goal is to analyze the full or joint pseudolikelihood in Eq. (2) instead of analyzing the full-conditionals in isolation. Analyzing the full-conditionals in isolation is common practice since it is fast. However, it leads to two potentially divergent parameter estimates for the associations and does not provide a coherent procedure for quantifying parameter uncertainty. By analyzing the joint pseudolikelihood, we can formulate a single prior distribution for the focal parameters to obtain a single posterior distribution that we can analyze in a meaningful way. The disadvantage of using the joint pseudolikelihood is its increased computational expense for some numerical procedures and the inability to analyze the full-conditionals in parallel. However, this increase in computational expense is negligible for the network sizes typically encountered in psychological applications.

The continuous spike-and-slab approach to select a network’s topology poses three critical challenges that we address in this paper. The first challenge that we address is the consistency of the structure selection procedure. In a recent analysis of covariate selection in linear regression, Narisetty and He (2014) showed that the continuous spike-and-slab approach is inconsistent if the hyperparameters are not correctly scaled. We extend this observation to the current structure selection problem^1^ and prove that a correct scaling of the hyperparameters leads to a consistent structure selection approach in an embedding with *p* fixed, *n* increasing. The second challenge that we address is the specification of tuning parameters. The effectiveness of the continuous spike-and-slab setup crucially depends on their specification. Unfortunately, objective methods to specify these parameters are currently unavailable, and tuning them is difficult and context dependent (e.g., George & McCulloch, 1997; O’Hara & Sillanpää, 2009). To overcome this issue, we develop a new procedure to automatically set the tuning parameters in such a way that we achieve a high specificity. The final challenge that we address is the practical exploration of the structure space $\mathcal {S}$. Even for relatively small networks, the structure space $\mathcal {S}$ can be vast, and exploring it poses a significant challenge. Moreover, even the most plausible structures have relatively small posterior probabilities and many similar structures exist (George, 1999). As a result, valuable computational effort is wasted on relatively uninteresting structures and it is difficult to estimate their probabilities with reasonable precision. To overcome this issue, we propose a novel, two-step approach. We first employ a deterministic estimation approach (Ročková & George, 2014), utilizing an expectation-maximization (EM; Dempster, Laird, & Rubin, 1977) variant of the continuous spike-and-slab approach to screen for a subset of promising edges. We then use a stochastic estimation approach (George & McCulloch, 1993), utilizing a Gibbs sampling (Geman & Geman, 1984) variant to explore the structure space instantiated by these promising edges. In sum, we propose a coherent Bayesian methodology for structure selection for the Ising model. The freely available R package rbinnet implements the proposed methods.^2^

The remainder of this paper is organized as follows. After this introduction, we first specify our Bayesian model, i.e., we discuss the pseudolikelihood and prior setup. Then, we analyze the consistency of our spike-and-slab approach for structure selection and show that it is consistent if suitably scaled. We wrap up the blueprint of our Bayesian model with the objective specification of hyperparameters for our spike-and-slab setup. We then present an EM and a Gibbs implementation of our Bayesian structure selection setup used for edge screening and structure selection, respectively. In our suite of Bayesian tools, edge screening most closely resembles eLasso, and we will compare the performance of these two methods in a series of simulations. Finally, we present a full analysis of data on alcohol abuse and major depressive disorders from the National Survey on Drug Use and Health. As far as we know, these two disorders have not been analyzed on a symptom level together in a network approach.

## Bayesian Model Specification

The setup of any Bayesian model comprises two parts: The likelihood of the model’s parameters and their prior distributions. We start with the likelihood dictated by the Ising model, and the pseudolikelihood approach that we adopt to circumvent the computational intractability of the full Ising model. We follow-up with the specification of prior distributions for the Ising model’s parameters, tying George and McCulloch’s (1993) continuous spike-and-slab prior setup for edge selection.

### The Ising Model Pseudolikelihood

In this paper, we will adopt the pseudolikelihood approach of Besag (1975), as presented in Eq. (2). We will furthermore assume that the observations are independent and identically distributed, such that the full pseudolikelihood becomes

$$\begin{aligned} p^*(\mathbf {X}\mid \varvec{\mu }\text {, }\varvec{\Sigma }) = \prod _{v=1}^n p^*(\mathbf {x}_v\mid \varvec{\mu }\text {, }\varvec{\Sigma }), \end{aligned}$$

where $\mathbf {X} = (\mathbf {x}_1^\mathsf{T}\text {, }\dots \text {, }\mathbf {x}^\mathsf{T}_n)^\mathsf{T}$, and we have adopted *v* to index the *n* independent and identically distributed observations. Both maximum pseudolikelihood and Bayesian pseudoposterior estimates are consistent as *n* increases (e.g., Arnold & Strauss, 1991; Geys, Molenberghs, & Ryan, 2007; Miller, 2019) and can consistently uncover the unknown graph structure of the full Ising model (Barber & Drton, 2015; Csiszár & Talata, 2006; Meinshausen & Bühlmann, 2006; Ravikumar et al., 2010). As a result, the pseudolikelihood has become an indispensable tool in the structure selection of Ising models.

### The Continuous Spike-and-Slab Prior Setup and Its Relation to Other Approaches

There are several ways to bring the indicator variables into our Bayesian model (e.g., Dellaportas, Forster, & Ntzoufras, 2002; George & McCulloch, 1993; Kuo & Mallick, 1998). O’Hara and Sillanpää 2009 and Consonni, Fouskakis, Liseo, and Ntzoufras Consonni et al. (2018) provide two recent overviews. One interesting approach was recently proposed by Pensar, Nyman, Niiranen, and Corander Pensar et al. (2017), which essentially uses the indicator variables to draw a Markov blanket in the full-conditional distributions of the Ising model and then, construct a marginal pseudolikelihood to select the network’s structure. A key aspect of their approach is that they formulated a Bayesian model on the individual pseudolikelihoods rather than the model’s parameters, and, using a few simplifying assumptions, they were able to derive analytic expressions for the marginal pseudolikelihoods. Unfortunately, this also required treating the pairwise associations as nuisance parameters. As a result, inference on the model’s parameters remains out of reach, and, in addition, it is unclear how the priors on the pseudolikelihoods translate to the model’s parameters. We will take a different route, but a numerical comparison between our approach and that of Pensar et al. Pensar et al. (2017)—implemented in the R package |BDgraph| (R. Mohammadi & Wit, 2019)—can be found in the online appendix.

In this paper, we adopt the continuous spike and slab approach, which comprises two parts. First, a mixture of two zero-centered normal distributions is imposed on the focal parameters. Here, the focal parameters are the pairwise associations $\sigma _{ij}$. The indicator variables are then used to distinguish between the two mixture components, and thus, the prior distribution on the focal parameters becomes

$$\begin{aligned} \sigma _{ij} \mid \gamma _{ij} \sim (1-\gamma _{ij})\, \mathcal {N}(0\text {, }\nu _0) + \gamma _{ij}\, \mathcal {N}(0\text {, }\nu _1), \end{aligned}$$

where $\mathcal {N}(0\text {, }\nu )$ denotes the normal distribution with a mean equal to zero and a variance equal to $\nu$. A small but positive variance $\nu _0 > 0$ is assigned to the component that is associated with $\gamma _{ij} = 0$ to encourage the exclusion of negligible nonzero values, and a large variance $\nu _1>> \nu _0$ is assigned to the component associated with $\gamma _{ij}=1$ to accommodate all plausible values of the interaction. The continuous spike-and-slab approach is a computationally convenient alternative to the discontinuous spike-and-slab approach that is common in model selection.

In the discontinuous spike-and-slab approach, the continuous spike distribution is replaced with a Dirac delta measure at zero. In other words, the association is set to zero for structures in which the relation is absent. The discontinuous spike-and-slab setup is popular in structure selection for Gaussian graphical models (GGMs) (GGMs; e.g., Carvalho & Scott, 2009; A. Mohammadi & Wit, 2015) and generalizations such as the copula GGM for binary and categorical variables (e.g., Dobra & Lenkoski, 2011) and the multivariate probit model for binary variables (e.g., Talhouk, Doucet, & Murphy, 2012)—variants of which are also implemented in the R packages |BDgraph| (R. Mohammadi & Wit, 2019) and |BGGM| (Williams & Mulder, 2020b). For the GGM and its generalizations, the slab priors are assigned to the inverse-covariance or precision matrix (i.e., the matrix of partial correlations) and thus, often use Wishart-type priors rather than the normal distribution that we propose for the Ising model’s associations.

The upside of using discontinuous over continuous spike-and-slab priors is that one only needs to consider the slab prior specification and that structure selection consistency is more easily attained. The downside, however, is that for models such as the Ising model, we run into severe computational challenges. The EM and Gibbs solutions that we advocate in this paper would not work for the Ising model if we would use the discontinuous spike-and-slab setup. The primary reason for this is that one cannot analytically integrate out the focal parameters for updating the edge indicators. Pensar et al. (2017) were able to derive their analytic solutions by stipulating the Ising model’s pseudolikelihood as the focal parameter and assuming orthogonality between different full-conditions. The continuous spike-and-slab approach proposed in this paper does not require an analytic integration of effects from the likelihood and is thus opportune to use in combination with the Ising model. Wang (2015) also applied it to edge-selection for the GGM, which is implemented in the R package |ssgraph| (R. Mohammadi, 2020).

The second part of our spike-and-slab approach is the specification of a prior distribution on the selection variables. Here, the selection variables are *a priori* modeled as i.i.d. Bernoulli$(\theta )$ variables, which implies the following prior distribution on the structures $\varvec{\gamma }_s$,

3 $$\begin{aligned} p(\varvec{\gamma }_s) = \theta ^{\gamma _{s++}}\,(1-\theta )^{{p\atopwithdelims (){2}}-\gamma _{s++}}, \end{aligned}$$

where $\gamma _{s++} = \sum _{i=1}^{p-1}\sum _{j=i+1}^p\gamma _{sij}$, with $\gamma _{ij} = \gamma _{ji}$. Once the hyperparameters $\nu _0$, $\nu _1$ and $\theta$ are set, and the nuisance parameters are assigned a prior distribution, the posterior structure probabilities can then be estimated using, for example, a Gibbs sampler (Geman & Geman, 1984; George & McCulloch, 1993). We stipulate independent standard-normal prior distributions on the nuisance parameters $\varvec{\mu }$ and make the objective specification of hyperparameters the topic of the ensuing sections.

## Structure Selection Consistency

In this section, we analyze posterior selection consistency, the ability of our method to determine the correct network structure consistently. As alluded to in the introduction, selection consistency using George and McCulloch’s spike and slab approach crucially depends on the hyperparameters $\nu _0$ and $\nu _1$. Unfortunately, fixing these parameters does not guarantee that our structure selection procedure is consistent. Narisetty and He (2014) showed that the use of fixed constants may lead to an inconsistent selection procedure in the context of linear regression. Below, we will demonstrate that this is also the case in the context of structure selection for Ising models. However, we will also show that our selection approach is consistent if the spike variance $\nu _0$ shrinks as a function of *n*.^3^ Narisetty and He presented a similar result for linear regression.

We first work out the concepts relevant for selection consistency, such as the posterior structure probability, and derive an approximate Bayes factor that is useful for the large-sample analysis. Then, we analyze the case with fixed hyperparameters and show that the selection procedure is inconsistent for fixed *p*, increasing *n*. Finally, we analyze the situation where the spike variance shrinks with *n* and show that this shrinking hyperparameter setup leads to a consistent selection procedure for fixed *p*, increasing *n*.

### Selection Consistency

We assume that the true structure *t* is in the set $\mathcal {S}$.^4^ We quantify our uncertainty in selecting a structure *s*, $s\in \mathcal {S}$, using the posterior structure probability

$$\begin{aligned} p(\varvec{\gamma }_s \mid \varvec{X}) = \frac{ p^*(\mathbf {X} \mid \varvec{\gamma }_s)\, p(\varvec{\gamma }_s)}{ \sum _{s\in \mathcal {S}} p^*(\mathbf {X} \mid \varvec{\gamma }_s)\, p(\varvec{\gamma }_s)} = \frac{\text {BF}^*_{st}\, \text {o}_{st}}{1 + \sum _{u \in \mathcal {S}_{\setminus t}} \text {BF}^*_{ut}\, \text {o}_{ut}}, \end{aligned}$$

where $p^*(\mathbf {X} \mid \varvec{\gamma }_s)$ denotes the integrated pseudolikelihood for the structure *s*, $\text {BF}^*_{st}$ the Bayes factor pitting structure *s* against the correct structure *t*, and $\text {o}_{st}$ denotes the prior model odds of the two structures. Selection consistency requires us to show that the posterior structure probabilities $p(\varvec{\gamma }_s \mid \mathbf {X})$ tend to zero for structures $s \ne t$, and that $p(\varvec{\gamma }_t \mid \mathbf {X})$ tends to one as the sample size grows. This is equivalent to showing that the Bayes factors $\text {BF}_{st}$ tend to zero for structures $s \ne t$. Unfortunately, analytic expressions for the Bayes factors are currently unavailable. To come to a workable expression for the Bayes factor, we first redefine it in terms of the expected prior odds under the correct posterior distribution

$$\begin{aligned} \text {BF}_{st}^*&= \frac{p^*(\mathbf {X} \mid \varvec{\gamma }_s)}{p^*(\mathbf {X} \mid \varvec{\gamma }_t)}\\&= \frac{\int \int p^*(\mathbf {X} \mid \varvec{\mu }\text {, }\varvec{\Sigma })\, p(\varvec{\mu })\, p(\varvec{\Sigma }\mid \varvec{\gamma }_s) \,\text {d}\varvec{\Sigma }\,\text {d}\varvec{\mu }}{\int \int p^*(\mathbf {X} \mid \varvec{\mu }\text {, }\varvec{\Sigma })\, p(\varvec{\mu })\, p(\varvec{\Sigma }\mid \varvec{\gamma }_t) \,\text {d}\varvec{\Sigma }\,\text {d}\varvec{\mu }}\\&= \int \int \frac{p^*(\mathbf {X} \mid \varvec{\mu }\text {, }\varvec{\Sigma })\, p(\varvec{\mu })\, p(\varvec{\Sigma }\mid \varvec{\gamma }_s)}{p^*(\mathbf {X} \mid \varvec{\mu }\text {, }\varvec{\Sigma })\, p(\varvec{\mu })\, p(\varvec{\Sigma }\mid \varvec{\gamma }_t)}\\&\text {. }\times \frac{ p^*(\mathbf {X} \mid \varvec{\mu }\text {, }\varvec{\Sigma })\, p(\varvec{\mu })\, p(\varvec{\Sigma }\mid \varvec{\gamma }_t) }{\int \int p^*(\mathbf {X} \mid \varvec{\mu }\text {, }\varvec{\Sigma })\, p(\varvec{\mu })\, p(\varvec{\Sigma }\mid \varvec{\gamma }_t) \,\text {d}\varvec{\Sigma }\,\text {d}\varvec{\mu }}\,\text {d}\varvec{\Sigma }\,\text {d}\varvec{\mu }\\&= \mathbb {E}^*\left( \left. \frac{p(\varvec{\Sigma }\mid \varvec{\gamma }_s)}{p(\varvec{\Sigma }\mid \varvec{\gamma }_t)}\text { }\right| \text { }\mathbf {X}\text {, }\varvec{\gamma }_t\right) \\&= \mathbb {E}^*\left( \left. \prod _{i=1}^{p-1}\prod _{j=i+1}^p \frac{p({\sigma }_{ij} \mid \gamma _{sij})}{p({\sigma }_{ij} \mid \gamma _{tij})} \text { }\right| \text { }\mathbf {X}\text {, }\varvec{\gamma }_t\right) , \end{aligned}$$

which is the posterior expectation of the ratio of the prior distributions of $\varvec{\Sigma }$ for the two models, *s* and *t*, under the correct structure specification $\varvec{\gamma }_t$. This is a convenient representation, as we only have to consider the pseudoposterior distribution under the correct network structure. Observe that this representation also holds when the full Ising likelihood is used, except that in the latter case the Bayes factor $\text {BF}_{st}$ is expressed as the expected prior odds w.r.t the posterior distribution and not the pseudoposterior distribution. For a fixed network of *p* variables, the posterior distribution can be accurately approximated with a normal distribution as *n* becomes large (see, for instance, Miller, 2019, Theorem 6.2), and the same holds for the pseudoposterior distribution (see, for instance, Miller, 2019, Theorems 3.2 and 7.3). To come to a workable expression of the Bayes factor we approximate the pseudoposterior with a normal distribution (i.e., a Laplace approximation), which leads to the following first-order approximation of the Bayes factor (Tierney, Kass, & Kadane, 1989, Eq. 2.6),

4 $$\begin{aligned} \text {BF}^*_{st}&= \prod _{i=1}^{p-1}\prod _{j=i+1}^p \frac{p(\hat{\sigma }_{ij} \mid \gamma _{sij})}{p(\hat{\sigma }_{ij} \mid \gamma _{tij})} \left[ 1+\mathcal {O}(n^{-1})\right] \nonumber \\&\approx \prod _{i=1}^{p-1}\prod _{j=i+1}^p \frac{p(\hat{\sigma }_{ij} \mid \gamma _{sij})}{p(\hat{\sigma }_{ij} \mid \gamma _{tij})}\nonumber \\&=\prod _{i=1}^{p-1}\prod _{j=i+1}^p \left( \sqrt{\frac{\nu _0}{\nu _1}}\, \exp \left( \hat{\sigma }_{ij}^2\, \frac{\nu _1-\nu _0}{2\nu _1\nu _0}\right) \right) ^{\gamma _{sij}-\gamma _{tij}} \end{aligned}$$

where $\widehat{\varvec{\Sigma }} = [\hat{\sigma }_{ij}]$ is the mode of $p^*(\varvec{\Sigma }\text {, }\varvec{\mu }\mid \mathbf {X}\text {, }\varvec{\gamma }_t)$, or $p(\varvec{\Sigma }\text {, }\varvec{\mu }\mid \mathbf {X}\text {, }\varvec{\gamma }_t)$ if the full Ising likelihood is used. Tierney et al. (1989) show that the error of the first-order approximation—the rest term $\mathcal {O}(n^{-1})$—is of order 1/*n*. Since the pseudoposterior is consistent (c.f., Miller, 2019, Theorem 7.3), the Bayes factor using the pseudolikelihood and the full likelihood will converge to the same number.

We will show next that the approximate Bayes factors $\text {BF}^*_{st}$, for $s \ne t$, do not shrink to zero with the three hyperparameters fixed, but do shrink to zero if $\nu _0$ shrinks to zero at a rate $n^{-1}$. The approximate Bayes factor comprises a product of the edge specific functions

$$\begin{aligned} f_{ij} = \left( \sqrt{\frac{\nu _0}{\nu _1}}\, \exp \left( \hat{\sigma }_{ij}^2\, \frac{\nu _1-\nu _0}{2\nu _1\nu _0}\right) \right) ^{\gamma _{s\text {, }ij}-\gamma _{t\text {, }ij}} \ge 0 \end{aligned}$$

which consists of two parts: The selection variables $\gamma _{s\text {, }ij}$ and $\gamma _{t\text {, }ij}$ that inform about the differences in edge composition of structures *s* and *t*, and the function $\sqrt{\nu _0/\nu _1}\exp \left( \hat{\sigma }_{ij}(\nu _1-\nu _0)/2\nu _1\nu _0\right)$ that weighs in the contribution of the pseudoposterior. The edge specific function $f_{ij}$ is equal to one if the edge is present in both structures, or is absent from both structures, since then $\gamma _{t\text {, }ij} - \gamma _{s\text {, }ij}=0$. We therefore only have to consider what happens to the function $f_{ij}$ for cases where $\gamma _{s\text {, }ij} \ne \gamma _{t\text {, }ij}$.

#### The Fixed Hyperparameter Case

If $\gamma _{t\text {, }ij}$ is equal to zero, and $\gamma _{s\text {, }ij}$ is equal to one, the correct value for the interaction parameter $\sigma _{ij}$ is zero, and we observe that

$$\begin{aligned}f_{ij} \overset{n}{\longrightarrow } \sqrt{\frac{\nu _0}{\nu _1}},\end{aligned}$$

which, even though it is smaller than one and signals a preference for structure *t*, does not converge to zero as it should if the structure selection procedure would be consistent. If $\gamma _{t\text {, }ij}$ is equal to one, and $\gamma _{s\text {, }ij}$ is equal to zero, on the other hand, such that $|\sigma _{ij}|>0$, we observe that

$$\begin{aligned} f_{ij} \overset{n}{\longrightarrow } \sqrt{\frac{\nu _1}{\nu _0}}\, \exp \left( -\sigma _{ij}^2\, \frac{\nu _1-\nu _0}{2\nu _1\nu _0}\right) , \end{aligned}$$

which does not converge to zero either. In fact, it may even signal a preference for the absence of the edge in structure *s*. These two observations indicate that the Bayes factors $\text {BF}^*_{st}$ do not converge to zero, and thus, the posterior probability $p(\varvec{\gamma }_t \mid \mathbf {X})$ does not converge to one. In sum, the proposed structure selection procedure is inconsistent in the case that the three hyperparameters are fixed.

#### The Shrinking Hyperparameter Case

We next consider the case where $\nu _0$ shrinks at a rate $n^{-1}$ and define $\nu _0 = \tfrac{\nu _1 \xi }{n}$. Here, $\xi$ is a fixed (positive) penalty parameter that allows us some flexibility to emphasize the distinction between the spike and slab components. If, in this case, $\gamma _{t\text {, }ij}$ is equal to one and $\gamma _{s\text {, }ij}$ is equal to zero, the function $f_{ij}$ is equal to

$$\begin{aligned} f_{ij} = \sqrt{\frac{n}{\xi }}\, \exp \left( -\hat{\sigma }_{ij}^2\, \frac{n-\xi }{2\nu _1\xi }\right) = \exp \left( \frac{1}{2}\log \left( \frac{n}{\xi }\right) - \hat{\sigma }_{ij}^2\frac{n-\xi }{2\nu _1\xi }\right) , \end{aligned}$$

where the first factor tends to infinity, and the second factor tends to zero. Because the second factor tends to zero faster than the first factor tends to infinity, their product, again, tends to zero, as it should. On the other hand, if $\gamma _{t\text {, }ij}$ is equal to zero, the function $f_{ij}$ becomes

$$\begin{aligned} \sqrt{\frac{\xi }{n}}\, \exp \left( \hat{\sigma }_{ij}^2\, \frac{n-\xi }{2\nu _1\xi }\right) , \end{aligned}$$

where the first factor tends to zero, and the second factor tends to one because $\sqrt{n}\hat{\sigma }_{ij}$ tends to zero ($\hat{\sigma } =\mathcal {O}_p(1/\sqrt{n})$). Therefore, $f_{ij}$ tends to zero, as it should. In sum, the structure selection procedure is consistent if $\nu _0$ shrinks at a rate of $n^{-1}$.

## Objective Prior Specification

We follow the results in the previous section, and set the spike variance to $\nu _0 = \tfrac{\nu _1\xi }{n}$, which leaves the specification of the slab variance $\nu _1$, the penalty parameter $\xi$, and the prior inclusion probability to complete our Bayesian model blueprint. We first discuss a default setting for the spike and slab variances, i.e., the specification of $\nu _1$ and $\xi$. We then discuss two options for the prior inclusion probabilities that we adopt in this paper.

### Specification of the Spike and Slab Variances

One approach to find default values for the slab variance is to set it equal to *n* times the inverse of the Fisher information matrix $\mathcal {I}_{\Sigma }(\hat{\varvec{\Sigma }}\text {, }\hat{\varvec{\mu }})^{-1}$, which approximately gives the information about $\sigma _{ij}$ in a single observation, hence the name *unit information* (Kass & Wasserman, 1995). Kass and Wasserman 1995 showed that the logarithm of the Bayes factor—pitting one network structure against another—is approximately equal to the difference in Bayesian information criteria (BIC; Schwarz, 1978) of the two structures when we use unit information priors (see also, Raftery, 1999; Wagenmakers, 2007, for details). This result, combined with the fact that unit information priors can be automatically selected, makes them a popular approach in Bayesian variable selection. We follow the approach of Ntzoufras (2009), who achieved good results by setting the off-diagonal elements of $\mathcal {I}^{-1}$ to zero in the prior specification. This renders the spike-and-slab prior densities independent, and sets the slab variance to $\nu _{1\text {, }ij} = n \text {Var}(\hat{\sigma }_{ij})$.^5^ If we set the slab variance equal to the unit information, the spike variance is equal to $\nu _{0\text {, }ij}= \xi \, \text {Var}(\hat{\sigma }_{ij})$. Our structure selection procedure will still consistently select the correct structure, since $\nu _{0\text {, }ij}$ shrinks with rate *n* because $\text {Var}(\hat{\sigma }_{ij})$ does (e.g., Miller, 2019, Section 5.2).

**Fig. 1:**
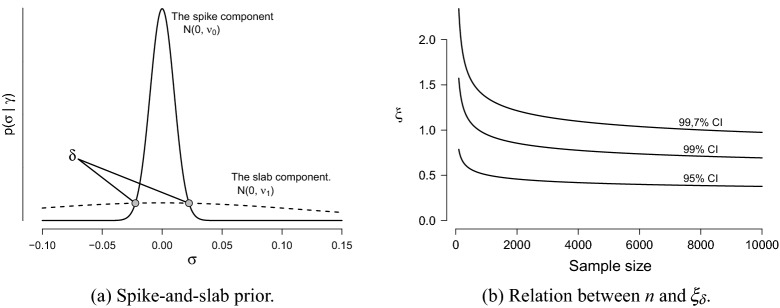
The left panel illustrates the spike-and-slab prior distribution and it’s intersection point $\delta$. The right panel illustrates the relationship between *n* and the $\xi _\delta$ value equating the intersection points $\pm \delta$ to three different credible intervals.

The spike-and-slab parameters are specified up to the constant $\xi$, which acts as a penalty parameter on the inclusion and exclusion of effects in the spike-and-slab prior. Larger values for $\xi$ increase the overlap between the spike-and-slab components and consequently, make it more likely that an effect is excluded, i.e., ends up in the spike component. It is the opposite case for smaller values. It is thus absolutely crucial to find a good value for this penalty. We wish to specify the tuning parameter $\xi$ such that the performance of our edge selection approach is similar to that of eLasso. To that aim, we introduce an automated procedure to specify the tuning parameter such that the corresponding continuous spike-and-slab setup is geared towards achieving a high specificity, or low type-1 error, similar to eLasso. The idea that we pursue here is to set the intersection of the spike-and-slab components equal to an approximate credible interval about zero. The left panel in Fig. 1 illustrates the idea.

George and McCulloch (1993) show that the two densities intersect at

$$\begin{aligned} |\delta | = \sqrt{\nu _1\frac{\log \left( \frac{\nu _1}{ \nu _0}\right) }{\frac{\nu _1}{\nu _0}-1}}. \end{aligned}$$

If we fill in our definitions for the spike and slab variances, the expression for $|\delta |$ boils down to

5 $$\begin{aligned} |\delta | = \sqrt{n \, \text {Var}(\hat{\sigma }_{ij})\frac{\log \left( \frac{n}{ \xi }\right) }{\frac{n}{\xi }-1}}, \end{aligned}$$

Where George and McCulloch (1993) discuss the subjective specification of $\delta$, we explore its automatic specification by matching it to the approximate credible interval. We first determine the range of parameter values $(-|\delta |\text {, }|\delta |)$ considered to be insignificant, and then select the value of $\xi$ such that the spike and slab components intersect at $\pm |\delta |$. When *n* is sufficiently large, the pseudoposterior distribution of an association parameter ${\sigma }_{ij}$ is approximately normal (Miller, 2019), and $\text {Var}(\hat{\sigma }_{ij})$ is its approximate variance. Thus, $(\hat{\sigma }_{ij}\,\pm \,3\sqrt{\text {Var}(\hat{\sigma }_{ij})})$ offers an approximate $99,7\%$ credible interval about the posterior mean $\hat{\sigma }_{ij}$. To set the variance of the spike distribution for negligible effects, it is opportune to use the interval $(\pm \,3\sqrt{\text {Var}(\hat{\sigma }_{ij})})$, which offers an approximate credible interval about zero, i.e., the credible interval assuming that the edge *i*–*j* should, in fact, be excluded from the model. Equating the expression for $|\delta |$ on the right side of Eq. (5) with $3\sqrt{\text {Var}(\hat{\sigma }_{ij})}$ gives:

6 $$\begin{aligned} \sqrt{n \frac{\log \left( \frac{n}{ \xi }\right) }{\frac{n}{\xi }-1}} = 3, \end{aligned}$$

which we can solve numerically to obtain a value for $\xi$. Observe that, by specifying $\delta$ in this particular way, the penalty parameter $\xi$ depends on the sample size but not the data or network’s size. We denote the value of the penalty parameter that matches the intersection $\delta$ to the credible interval with $\xi _\delta$. The relation between $\xi _\delta$ and sample size is illustrated in the right panel of Fig. 1.

### Specification of the Prior Inclusion Probability

Assuming that the correct structure is in $\mathcal {S}$, i.e., the $\mathcal {S}$-closed view of structure selection, a default choice to express ignorance or indifference between the structures in $\mathcal {S}$ is to stipulate a uniform prior distribution over the topologies in $\mathcal {S}$:

$$\begin{aligned} p(\varvec{\gamma }_s) = \frac{1}{|\mathcal {S}|}, \text { for }\varvec{\gamma }_s \in \mathcal {S}, \end{aligned}$$

where $|\mathcal {S}|$ denotes the cardinality of the structure space. Here, the uniform prior is equal to

$$\begin{aligned} p(\varvec{\gamma }_s) = 2^{-\tfrac{1}{2}p\,(p-1)}, \end{aligned}$$

and we can impose this prior on the structure space by fixing the prior inclusion probability $\theta$ in Eq. (3) to $\tfrac{1}{2}$. However, the uniform prior on the structure space does not take into account structural features of the models under consideration, such as sparsity. Various priors have been proposed as an alternative to accommodate these features (see Consonni et al., 2018, Section 3.6, for a detailed discussion). One particular issue inherent in structure comparisons is multiplicity, and Scott and Berger (2010) argue that the prior distribution should account for this. Consonni et al. (2018) show that stipulating a hyperprior on the prior inclusion probability $\theta$ accounts for multiplicity. In particular, they showed that the uniform hyperprior $\text {Beta}(1\text {, }1)$ leads to the following prior on the structure space

$$\begin{aligned} p(\varvec{\gamma }_s) = p(\varvec{\gamma }_s \mid \gamma _{s, ++} = c) \times p(\gamma _{s, ++} = c) = \frac{1}{{p\atopwithdelims ()2} + 1} \times \frac{1}{{{p\atopwithdelims ()2} \atopwithdelims ()\gamma _{s, ++}}}, \end{aligned}$$

where *c* denotes the complexity of structures, with $c \in (0\text {, }1\text {, }\dots \text {, } {p\atopwithdelims ()2})$, i.e., the number of edges in the topology. Thus, instead of a uniform prior on the structure space, the hierarchical prior stipulates a uniform prior on the structure’s complexity. As a result, it favors models that have a relatively extreme level of complexity, e.g., are densely connected or are sparsely connected.

**Fig. 2:**
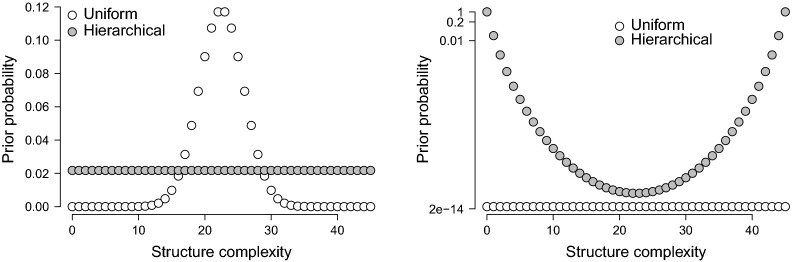
The left panel illustrates how the two prior distributions assign probabilities to structure complexity. The right panel illustrates how the two prior distributions assign probabilities to different structures with the same complexity. The prior probabilities in the right panel are shown on a log scale. For both panels, $p = 10$.

Figure 2 illustrates the different probabilities that the two distributions assign to structure complexity, a priori, and the probabilities they assign to structures that have the same complexity (shown on a log scale). The left panel of Fig. 2 shows that whereas the hierarchical prior is uniform on the complexity, the uniform prior is not and favors structures that have approximately half of the available edges. However, the right panel of Fig. 2 illustrates that the hierarchical prior emphasizes structures at the extremes of complexity. We will adopt both the uniform prior distribution on the structure space and the uniform prior distribution on the structure’s complexity, and analyze them further in the section on numerical illustrations. Based on Fig. 2, however, we expect that for small samples, the hierarchical prior will place much emphasis on extremely sparse structures, since our penalty selection approach already gears toward sparse solutions.

## Bayesian Edge Screening and Structure Selection for the Ising model

George and McCulloch (1993) proposed stochastic search variable selection (SSVS) as a principled approach to Bayesian variable selection. SSVS uses the spike-and-slab prior specification to emphasize the posterior probability of promising structures and Gibbs sampling to extract this information from the data at hand. The Gibbs sampler is a powerful tool for the exploration of the posterior distribution of potential network structures. However, since the structure space $\mathcal {S}$ can be quite large in practical settings, it might take a while to sufficiently explore the posterior distribution and produce reliable estimates of the posterior structure probabilities. We, therefore, wish to prune the structure space by selecting the promising edges before running the Gibbs sampler. We explore an EM variable selection approach for this initial edge screening and then, follow-up with an SSVS approach for structure selection on the set of promising edges.

### Edge Screening with EM Variable Selection

Ročková and George (2014) were the first to propose the use of EM for Bayesian variable selection, in combination with the spike-and-slab prior specification of George and McCulloch (1993), to covariate selection of linear models. The EM algorithm aims to find the posterior mode of the pseudoposterior distribution $p^*(\varvec{\Sigma }\text {, }\varvec{\mu }\text {, }\theta \mid \mathbf {X})$ and does this by iteratively maximizing the “complete data” pseudoposterior distribution $p^*(\varvec{\Sigma }\text {, }\varvec{\mu }\text {, }\theta \text {, }\varvec{\gamma }\mid \mathbf {X})$, treating the selection variables $\varvec{\gamma }$ as missing or latent variables. The algorithm alternates between two steps. In the expectation or E-step, we compute the expected log-pseudoposterior distribution, or Q-function,

$$\begin{aligned} \text {Q}&\left( \left. \varvec{\Sigma }\text {, }\varvec{\mu }\text {, }\theta \,\right| \, \varvec{\Sigma }^k\text {, }\theta ^k\right) = \mathbb {E}\left( \left. \ln p^*(\varvec{\Sigma }\text {, }\varvec{\mu }\text {, }\theta \text {, }\varvec{\gamma }\mid \mathbf {X})\,\right| \, \varvec{\Sigma }^k\text {, }\theta ^k\right) , \end{aligned}$$

with respect to posterior distribution of the latent variables $p(\varvec{\gamma } \mid \varvec{\Sigma }^k\text {, }\theta ^k)$, where $\varvec{\Sigma }^k$, and $\theta ^k$ denote the estimates in iteration *k*. The E-step is followed by a maximization or M-step in which we find the values $\varvec{\Sigma }^{k+1}$, $\varvec{\mu }^{k+1}$ and $\theta ^{k+1}$ that maximize the Q-function. The two steps are repeated until convergence.

The E-step of the EM algorithm involves expectations of the latent or missing variables, i.e., the vector of selection variables $\varvec{\gamma }$. Since the latent selection variables only operate in the spike-and-slab prior distributions, the derivation of the E-step will follow the derivation of Ročková and George (2014). For a complete treatment of EMVS, however, we include an analysis of both the E-step and the M-step in “Appendix A”. “Appendix A” also includes details about estimating the (asymptotic) posterior standard deviations from the EM output.

#### Edge Screening

The EM algorithm that we outlined in the previous section identifies a posterior mode $(\widehat{\varvec{\Sigma }}\text {, }\hat{\varvec{\mu }}\text {, }\hat{\theta })$, and we threshold the modal estimates to obtain a tightly matching network structure $\hat{\varvec{\gamma }}$. The idea of Ročková and George (2014) that we pursue here is that "large" interaction effect estimates define a set of promising edges, and we can thus prune edges that link to "small" interaction effect estimates. We define the structure $\hat{\varvec{\gamma }}$ that closely matches the modal estimates $(\widehat{\varvec{\Sigma }}\text {, }\hat{\varvec{\mu }}\text {, }\hat{\theta })$ to be the most probable structure $\varvec{\gamma }$ given the parameter values $({\varvec{\Sigma }}\text {, }{\varvec{\mu }}\text {, }{\theta }) = (\widehat{\varvec{\Sigma }}\text {, }\hat{\varvec{\mu }}\text {, }\hat{\theta })$, i.e.,

7 $$\begin{aligned} \hat{\varvec{\gamma }} = \arg \max _{\varvec{\gamma }} \, p(\varvec{\gamma } \mid \widehat{\varvec{\Sigma }}\text {, }\hat{\theta }). \end{aligned}$$

For our Bayesian model, the posterior inclusion probabilities for the different edges are conditionally independent, and the posterior inclusion probability for an edge *i*–*j* is given by

$$\begin{aligned} p(\gamma _{ij} \mid \hat{\sigma }_{ij}\text {, }\hat{\theta }) = \frac{p(\hat{\sigma }_{ij} \mid \gamma _{ij})\, p(\gamma _{ij} \mid \hat{\theta })}{\sum _{\Gamma _{ij}=\gamma _{ij}} p(\hat{\sigma }_{ij} \mid \gamma _{ij})\, p(\gamma _{ij} \mid \hat{\theta })}. \end{aligned}$$

Thus, we obtain $\hat{\varvec{\gamma }}$ from maximizing the inclusion and exclusion probabilities in Eq. (7), for each of the ${p\atopwithdelims ()2}$ edges, which means that

$$\begin{aligned} \hat{\gamma }_{ij} = 1 \, \Longleftrightarrow \, p(\gamma _{ij} = 1 \mid \hat{\sigma }_{ij}\text {, }\hat{\theta }) \ge 0.5, \end{aligned}$$

and we prune the edges for which $p(\gamma _{ij} = 0 \mid \hat{\sigma }_{ij}\text {, }\hat{\theta }) \ge 0.5$. This edge selection and pruning approach leads to a structure $\varvec{\gamma }$ that is a median probability model, as defined by Barbieri and Berger (2004) to be the structure comprising edges that have a posterior inclusion probability at or above a half.^6^ Ročková and George (2014) show that instead of selecting the structure $\hat{\varvec{\gamma }}$ based on the posterior inclusion probabilities, we may equivalently select it through thresholding the values of $\hat{\sigma }_{ij}$. Specifically,

8 $$\begin{aligned} \hat{\gamma }_{ij} = 1 \, \Longleftrightarrow \, |\hat{\sigma }_{ij}| \ge \sqrt{ 2 \, \log \left( \frac{1-\hat{\theta }}{\hat{\theta }} \, \sqrt{\frac{\nu _1}{\nu _0}}\right) \, \frac{\nu _1\nu _0}{\nu _1 - \nu _0}} = \sqrt{ 2 \, \log \left( \frac{1-\hat{\theta }}{\hat{\theta }} \, \sqrt{\frac{n}{\xi }}\right) \, \frac{n\text {Var}(\hat{\sigma }_{ij})\xi }{n - \xi }}. \end{aligned}$$

Such a connection between the magnitude of the modal estimates $\hat{\sigma }_{ij}$ and promising edges *i*–*j*, we envisioned from the beginning. Observe that, since $n\text {Var}(\hat{\sigma }_{ij})$ is the unit information, i.e., it is a constant, the right-most factor shrinks with *n*. Moreover, it shrinks much faster than that $\log (\sqrt{n})$ tends to infinity, such that the threshold moves to smaller values as *n* increase, as it should.

### Structure Selection with SSVS

The EMVS approach enables us to screen for a promising set of edges by locating a local posterior mode and pruning edges associated with small modal parameters. The structure $\varvec{\gamma }^\prime$ that comes out of this pruned edge set is a local median probability structure. We now wish to directly explore $p^*(\varvec{\gamma }\mid \mathbf {X})$, the pseudoposterior distribution of network structures, to find out if $\varvec{\gamma }^\prime$ is also the global median probability model, and if there are other promising structures for the data at hand. We do this using the stochastic search and variable selection (SSVS) approach of George and McCulloch (1993), which essentially combines the spike-and-slab prior setup with Gibbs sampling to produce a sequence

$$\begin{aligned} \varvec{\gamma }^{(0)}\text {, }\varvec{\gamma }^{(1)}\text {, }\varvec{\gamma }^{(2)}\text {, }\dots , \end{aligned}$$

which converges in distribution to samples from $\varvec{\gamma } \sim p(\varvec{\gamma } \mid \mathbf {X})$. We then shift our focus to structures $\varvec{\gamma }_s$ that occur frequently in the generated sequence, which are the structures that have a high posterior probability. We cut down the potentially large number of network structures that the Gibbs sampler needs to explore by applying SSVS only to the edges screened by EMVS.^7^

The Gibbs sampler operates by iteratively simulating values from the conditional distributions of (a subset of) the model parameters given the (other parameters and the) observed data. Unfortunately for us, the full-conditional distributions of our Bayesian model are not available in closed form, as the normal prior distributions that we have specified are not conjugate to the pseudolikelihood. However, since the pseudolikelihood comprises a sequence of logistic regressions, we can use the data-augmentation strategy that was proposed by Polson, Scott, and Windle (2013a) to facilitate a simple Gibbs sampling approach, with full-conditionals that are easy to sample from. A similar approach to the Ising model’s pseudolikelihood was considered by Donner and Opper (2017). Here, we extend this idea to SSVS for the Ising model.

Polson et al. (2013a) proposed an ingenious data augmentation strategy based on the identity

$$\begin{aligned} \frac{\left( e^{\psi }\right) ^a}{\left( 1+e^{\psi }\right) ^b} = \frac{1}{2^b} e^{\left( a-b/2\right) \,\psi } \int _{\mathbb {R}^+} e^{-\frac{1}{2}\omega \,\psi ^2} \, p(\omega ) \, \text {d}\omega , \end{aligned}$$

where $p(\omega )$ is a Pólya–Gamma distribution. A key aspect of this augmentation strategy is that it relates the logistic function of a parameter $\psi$ on the left to something that is proportional to a normal distribution on the right. Since our prior distributions are all (conditionally) normal, and the normal distribution is its own conjugate, the data-augmented full-conditionals will all be normal. To wit, applied to the pseudolikelihood in Eq. (2), we find

$$\begin{aligned}&\prod _{i=1}^pp^*(x_i \mid \mathbf {x}^{(i)}\text {, } \varvec{\mu }\text {, }\varvec{\Sigma }) \\&\quad =\frac{1}{2^p}\,\prod _{i=1}^p \,\int _{\mathbb {R}_+}\,e^{\left[ x_i-1/2\right] \left[ \mu _{i} + \sum _{j \ne i}\sigma _{ij}x_j\right] -\frac{1}{2}\omega _i\left[ \mu _{i} + \sum _{j \ne i}\sigma _{ij}x_j\right] ^2}\, p(\omega _i)\,\text {d}\omega _i, \end{aligned}$$

and with normal prior distributions for the pseudolikelihoods parameters we readily find normal full-conditional distributions when we condition on the augmented variables $\varvec{\omega }$. Another important aspect of the augmentation strategy is that the conditional distribution of the augmented variables $\omega$ given the pseudolikelihood parameters and the observed data $\mathbf {X}$ is again a Pólya-Gamma distribution. Polson et al. (2013a) and Windle, Polson, and Scott (2014) provide efficient rejection algorithms to simulate from this distribution.

With the Pólya-Gamma augmentation strategy in place, the Gibbs sampler iterates between five steps, which are detailed in “Appendix C”. The Gibbs output allows us to estimate a number of important quantities. For example, the posterior structure probabilities can be estimated as

$$\begin{aligned} p(\varvec{\gamma }_s \mid \mathbf {X}) \approx \frac{1}{R}\, \sum _{r=1}^R I(\varvec{\gamma }^{(r)} = \varvec{\gamma }_s), \end{aligned}$$

where $I(\cdot )$ is an indicator function that is equal to one if its conditions are satisfied and equal to zero otherwise, and the (global) posterior inclusion probabilities as,

$$\begin{aligned} p(\gamma _{ij} = 1 \mid \mathbf {X}) \approx \frac{1}{R}\, \sum _{r=1}^R \gamma _{ij}^{(r)}, \end{aligned}$$

were $\varvec{\gamma }^{(r)}$, for $r = 1, \text {, }\dots \text {, }R$, denotes *R* iterates of the Gibbs sampler. In a similar way, one can compute quantities related to the model-averaged posterior distribution of the model parameters, e.g.,

$$\begin{aligned} p(\varvec{\mu }\text {, }\varvec{\Sigma } \mid \mathbf {X}) = \sum _{\varvec{\gamma }_s\in \mathcal {S}} p(\varvec{\mu }\text {, }\varvec{\Sigma } \mid \varvec{\gamma }_s\text {, }\varvec{X}) p(\varvec{\gamma }_s \mid \mathbf {X}), \end{aligned}$$

or any of its marginals. In sum, the Gibbs sampler grants us the full Bayesian experience.

## Numerical Illustrations

In this section, we will focus on a comparison of our procedures with eLasso. A comparison between our edge screening and structure selection approaches and the approach of Pensar et al. (2017)—as implemented in |BDraph| (R. Mohammadi & Wit, 2019)—can be found in the online appendix. We have also included model selection for the multivariate probit model (e.g., Talhouk et al., 2012)—as implemented in |BGGM| (Williams & Mulder, 2020b)—in that comparison. There were some small variations, but overall the three different approaches performed very similar in terms of edge detection.

### Edge Screening on Simulated Data with Sparse Topologies

The eLasso approach of van Borkulo et al. (2014) is the most popular method for analyzing Ising network models in psychology. We wish to find out how our EMVS approach stacks up against eLasso, and we, therefore, use the simulation setup of (van Borkulo et al. 2014) to compare both methods. Specifically, we focus on the simulations that lead to their Table 2, where an Erdős and Rényi (1960) model is used to generate underlying sparse topologies, and normal distributions are used to simulate the model parameters.^8^ In these simulations, we vary $\pi$, the probability of generating an edge between two variables, *p*, the number of variables, and *n*, the number of observations and generate 100 datasets for each combination of values for $\pi$, *p*, and *n*.

We analyze the simulated datasets using eLasso, using the default settings implemented in the IsingFit program (van Borkulo, Epskamp, & Robitzsch, 2016), i.e., the AND-rule and an EBIC penalty equal to 0.25. We also analyze the simulated datasets using EMVS in combination with the $\xi _\delta$ method and the uniform and hierarchical specifications of the prior structure probabilities. We follow van Borkulo et al. (2014) and express the quality of the estimated solution using its sensitivity and specificity. Sensitivity is the proportion of present edges that are recovered by the method,

$$\begin{aligned} \text {SEN} = \frac{\text {True positive}}{\text {True positive} + \text {False negative}}, \end{aligned}$$

i.e., the true positive rate. Specificity is equal to the proportion of absent edges that are correctly recovered,

$$\begin{aligned} \text {SPE} = \frac{\text {True negative}}{\text {True negative} + \text {False positive}}, \end{aligned}$$

i.e., the true negative rate. For eLasso, edge inclusion refers to a nonzero association estimate using the AND approach. For EMVS, it is taken to mean that the posterior inclusion probability exceeds 0.5.

Table 1 shows the result of these simulations for the eLasso method in the column labelled “eLasso” and are similar to the results reported in Table 2 in van Borkulo et al. (2014). The first thing to note about these results is that eLasso has a high true negative rate across all simulations. This was to be expected, as $l_1$-regularization gears towards edge exclusions, which is why it performs best in the sparse network settings considered here. Indeed, it’s specificity goes down as the networks become more densely connected (i.e., larger values of $\pi$). The true positive rate of eLasso is significantly worse than its specificity, especially for the smaller sample sizes. However, the sensitivity increases with sample size, which underscores earlier results that a larger sample size helps overcome the prior shrinkage effect of the lasso (e.g., Epskamp, Kruis, & Marsman, 2017).

**Table 1:** Sensitivity and specificity, as a measure of performance of eLasso and EMVS using either a uniform (U) or hierarchical prior (H), matching the spike and slab intersections to an approximate 99,7% credible interval.

*n*	*p*		$\pi = 0.1$			$\pi = 0.2$			$\pi = 0.3$		
			eLasso	$\xi _\delta$(U)	$\xi _\delta$(H)	eLasso	$\xi _\delta$(U)	$\xi _\delta$(H)	eLasso	$\xi _\delta$(U)	$\xi _\delta$(H)
100	10	SEN	.264	.221	.044	.233	.251	.027	.218	.216	.032
		SPE	.997	.991	1.000	.994	.994	1.000	.993	.990	1.000
	20	SEN	.165	.240	.009	.171	.180	.004	.182	.114	.003
		SPE	.998	.991	1.000	.997	.992	1.000	.991	.993	1.000
	30	SEN	.151	.202	.002	.140	.118	.001	.142	.048	.000
		SPE	.999	.992	1.000	.995	.993	1.000	.979	.995	1.000
500	10	SEN	.557	.608	.484	.593	.575	.504	.595	.529	.462
		SPE	.997	.996	1.000	.992	.994	1.000	.989	.996	.999
	20	SEN	.519	.558	.455	.542	.497	.388	.550	.411	.268
		SPE	.998	.996	1.000	.990	.997	1.000	.972	.996	1.000
	30	SEN	.520	.526	.380	.489	.388	.265	.368	.196	.091
		SPE	.998	.996	1.000	.985	.996	1.000	.954	.998	1.000
1,000	10	SEN	.697	.730	.633	.675	.685	.639	.699	.681	.608
		SPE	.997	.997	1.000	.989	.996	1.000	.985	.996	.999
	20	SEN	.643	.680	.598	.676	.630	.565	.657	.545	.464
		SPE	.996	.996	1.000	.987	.997	1.000	.964	.997	.999
	30	SEN	.655	.645	.570	.635	.517	.449	.431	.298	.206
		SPE	.997	.997	1.000	.980	.997	1.000	.957	.997	1.000
2,000	10	SEN	.783	.811	.727	.759	.807	.738	.789	.770	.735
		SPE	.998	.997	1.000	.995	.995	0.999	.984	.996	1.000
	20	SEN	.740	.790	.715	.784	.738	.697	.765	.657	.623
		SPE	.997	.996	1.000	.985	.996	.999	.960	.997	.999
	30	SEN	.748	.761	.700	.738	.665	.609	.598	.441	.353
		SPE	.996	.996	1.000	.976	.997	1.000	.940	.997	1.000

Next, we consider the performance of EMVS. The results for EMVS when the penalty $\xi$ is set to $\xi _\delta$, the penalty value for which the intersection of the spike and the slab components aligns with the $99,7\%$ approximate credible interval, c.f. Eq. (6), are shown in the columns labeled $\xi _\delta$ in Table 1. We analyzed the data using the uniform prior on the model space—$\xi _\delta$ (U)—and with the hierarchical model—$\xi _\delta$ (H). The first striking result is that the performance of the $\xi _\delta$ approach combined with a uniform prior on the structure space performs almost identical to eLasso, making it a valuable Bayesian alternative to the classical eLasso approach. Observe that the specificity equals the coverage probability of the credible interval for all but the smallest sample size. Thus, as one might expect, the coverage probability specified dictates the method’s type-1 error or specificity. The hierarchical prior on the structure space leads to an improvement to the already high specificity. For the smaller sample sizes, however, the method’s sensitivity is very low, suggesting that it is, perhaps, too conservative for settings with small sample sizes.

### Parameter Estimation on Simulated Data with Dense Topology

We continue with an illustration of the estimation of parameters and inclusion probabilities. For this analysis, we simulate data for $n = 20,000$ cases on a $p = 15$ variable network. The main effects were simulated from a Uniform$(-1\text {, }1)$ distribution, and the matrix of associations $\varvec{\Sigma }$ was set to $\mathbf {u}\mathbf {u}^\mathsf{T}$, where $\mathbf {u}$ is a *p*-dimensional vector of Uniform$(-\tfrac{1}{2}\text {, }\tfrac{1}{2})$ variables, such that the elements in $\varvec{\Sigma }$ lie between $-\tfrac{1}{4}$ and $\tfrac{1}{4}$ and concentrate around zero. Observe that, in principle, this is a densely connected network as all edges have a nonzero value, although most effects will be very small and negligible. A second data set of $n=2,000$ cases was used to compare the performance across different sample sizes.

**Fig. 3:**
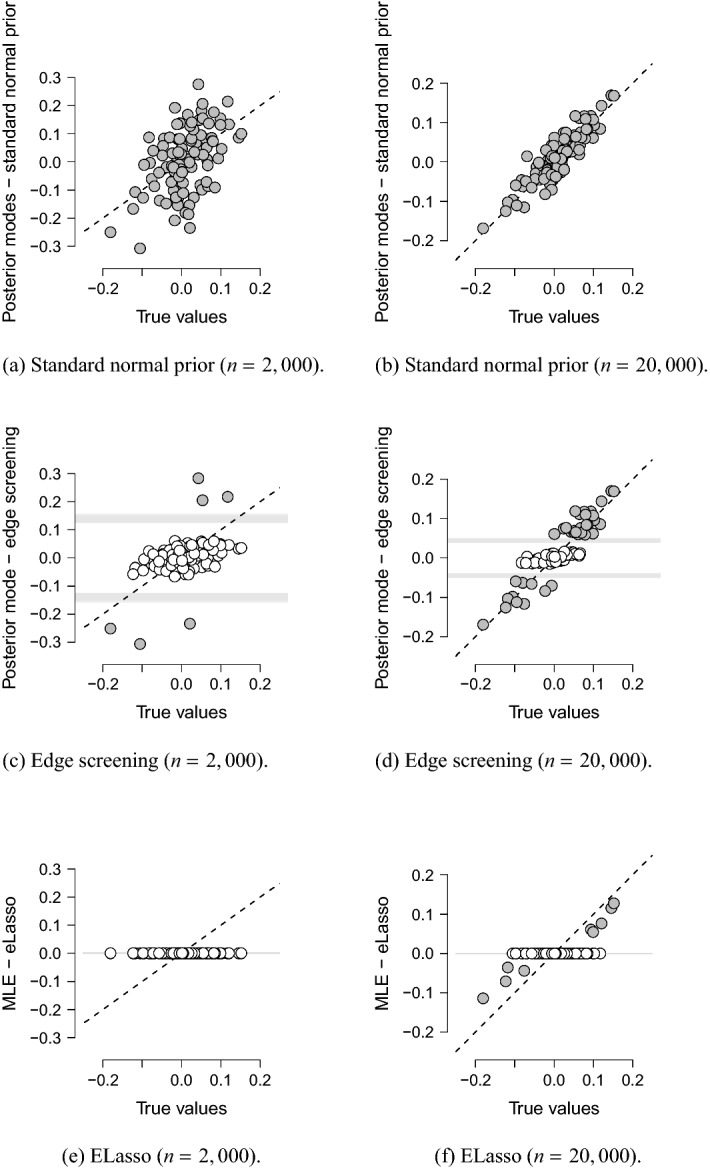
The posterior modes of the association parameters using a standard normal prior are shown in the top two panels, and the posterior modes of the associations using our spike-and-slab prior setup, i.e., edge screening, are shown in the middle two panels. The horizontal gray lines in (**c**) and (**d**) reveal the thresholds from Eq. (8). The bottom two panels show the maximum pseudolikelihood estimates produced by eLasso. The dashed lines are the bisection lines.

Figure 3 shows the posterior mode estimates for the two sample sizes using a standard normal prior distribution in Panels (a) and (b) and using our spike-and-slab setup, i.e., edge screening, in Panels (c) and (d). Observe that the effects are relatively small, and thus, many observations are needed to retrieve reasonable estimates (Panels (a) and (b)). We, therefore, cull considerably more of the effects in the edge screening step for the smaller sample size than for the larger sample size (white dots indicate culled associations in Panels (c) and (d)). The horizontal gray lines in Panels (c) and (d) reveal the spike-and-slab intersections for the different associations (there are 210 different lines, which all lie very close to each other), the thresholds from Eq. (8). Effects that lie in between the two intersection points end up in the spike (not selected; white dots); otherwise, they end up in the slab (selected; gray dots). Note that the intersections points lie closer to each other for the larger sample size, as expected. Panels (e) and (f) show the maximum pseudolikelihood estimates for eLasso, subject to the $l_1$ constraint, which selects considerably fewer effects for the larger sample size, and a substantial shrinkage effect on the associations.^9^

**Fig. 4:**
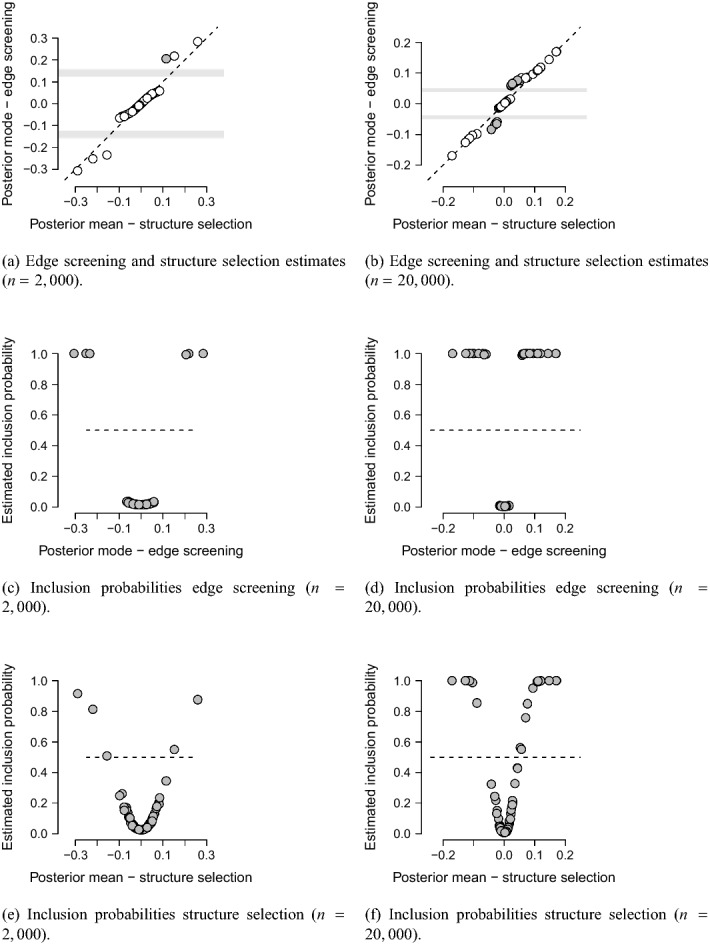
The top two panels show scatterplots of the posterior means and posterior modes of the association parameters that were obtained from our structure selection and edge screening procedures, respectively. The gray points are points of disagreement. The middle two panels show the edge screening inclusion probabilities and the bottom two panels the structure selection inclusion probabilities. The dashed lines are the bisection lines.

Figure 4 illustrates the various shrinkage effects in edge screening using EM and structure selection using the Gibbs sampler. Panels (a) and (b), for example, show that the procedures produce point estimates that are close to each other. Still, there is also variation between the two methods, especially around the spike and slab intersection lines. Although we did not show it here, the posterior estimates from EM and the Gibbs sampler were identical when we used a standard normal prior distribution instead of our spike-and-slab setup. These observations suggest that the differences gleaned from Panels (a) and (b) come from the fact that the edge screening procedure optimizes the vector of inclusion variables with EM while the structure selection procedure averages over them in the Gibbs sampler. These differences become even more apparent when we compare the inclusion probabilities they estimate. Panels (c) and (d) show the inclusion probabilities against the posterior mode estimates for the edge screening approach, and Panels (e) and (f) show the inclusion probabilities against the posterior mean estimates for the structure selection procedure. Whereas the inclusion probabilities lie close to zero or one for the EM approach, they show a much smoother relation for the Gibbs sampling approach. The ability to estimate inclusion probabilities that are close to one or zero is called separation, and it is clear that the EM approach shows a better separation than the Gibbs approach. But the spike-and-slab Gibbs sampling approach, i.e., SSVS, already shows excellent separation compared to other methods (e.g., O’Hara & Sillanpää, 2009). Even though the edge screening approach shows better separation, it is also more liberal, as it includes more effects into the model than the structure selection procedure does. Panels (a) and (b) indicate these points in gray in Panels (a) and (b).

## Network Analysis of Alcohol Use Disorder and Depression Data

For an empirical illustration of our Bayesian methods, we assess the relationship between symptoms of alcohol use disorder (AUD) and major depressive disorder (MDD) using data from the National Survey on Drug Use and Health (NSDUH; United States Department of Health and Human Services, 2016). The NSDUH is an American population study on tobacco, alcohol, and drug use, and mental health issues in the USA. The goal of the NSDUH is to provide accurate estimates on current patterns of substance abuse and its consequences for mental health. The survey is conducted in all 50 states, aiming at a sample of 70,000 individuals; participants have to be above the age of 12 and are randomly selected based on household addresses. We focus on the data on alcohol use and depression obtained in 2014.

The 2014 data comprises 55,271 participants. We exclude participants below the age of 18, that never drank alcohol, or that did not drink alcohol on more than six occasions in the past year. The final data analyzed here comprises 26,571 participants.

We included the seven items related to the DSM-V (American Psychiatric Association, 2013) criteria for AUD, and the nine symptoms in the NSDUH survey data comprising the DSM-V criteria for MDD in our analysis. The NSDUH derives the MDD symptoms from survey items formulated in a skip-structure. In this setup, participants are allowed to skip certain items based on the answers they provide. Therefore, some specifics of symptoms are not assessed for participants, which may cause more absence scores for symptoms or problems than is the case.

In our analyses below, we first screen the network for promising edges, and then select plausible structures from the structure space instantiated by the set of promising edges. We will also perform structure selection without this initial pruning to illustrate the necessity of the edge screening step.

### Edge Screening

**Fig. 5:**
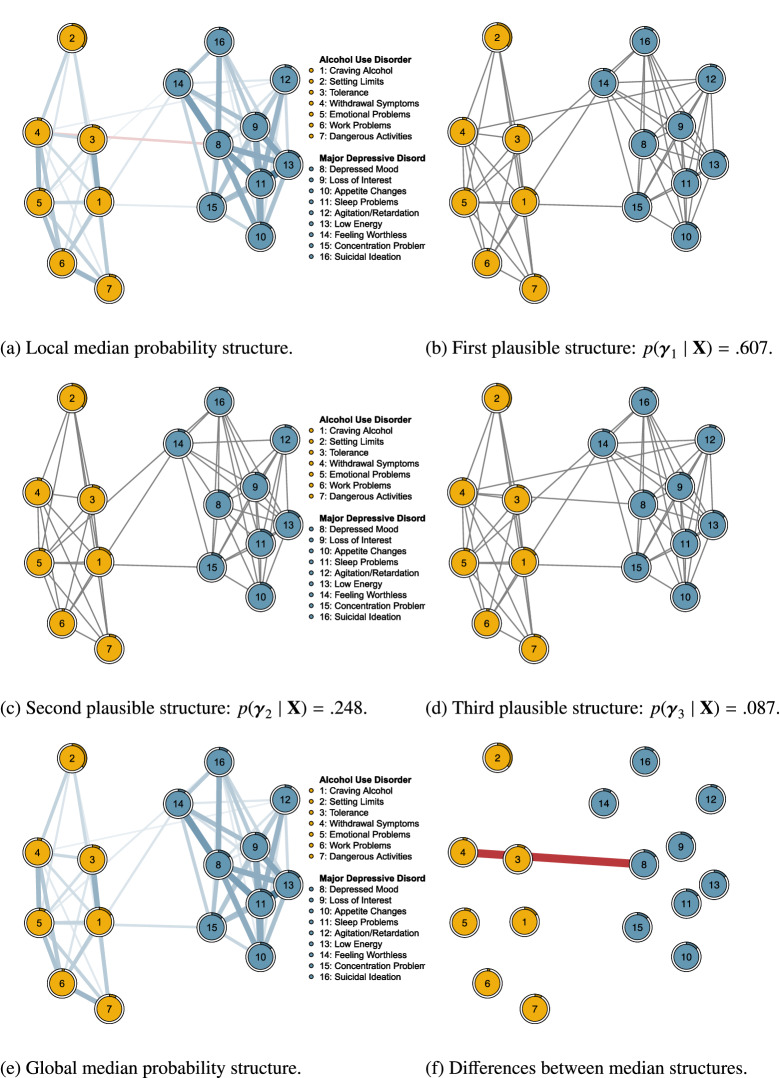
Edge screening and structure selection for NSDUH data. **a** indicates the network generated by the promising edges identified by edge screening; the local median probability structure. **b**–**d** indicate the three (most) plausible structures identified by structure selection on the pruned space. **e** indicates the global median probability structure, and **f** indicates the difference between the two median probability structures. The network plots are produced using the R package qgraph (Epskamp, Cramer, Waldorp, Schmittmann, & Borsboom, 2012).

In total, there were $p = 16$ variables, and ${16\atopwithdelims ()2} = 120$ associations or possible edges to consider. We ran the edge screening procedure using EMVS on the selected NSDUH data. The EMVS setup with a uniform prior on the structure space selected the same edges as the EMVS setup with a uniform prior on structure complexity. We continue here using the results from the former. The edge screening procedure identified 62 promising edges, pruning almost half of the available connections. Edge screening using a uniform prior distribution on structure complexity gave the same results. The eLasso method identified 61 edges, three of which were not identified by our edge screening procedure. There were four edges identified by our edge screening procedure, that were not identified by eLasso. Figure 5a shows the network generated by the screened edges, where blue edges constitute positive associations, and red edges constitute negative associations.

We glean several important observations from Fig. 5a. First, with 33 out of ${9\atopwithdelims ()2} = 36$ possible connections between its nine symptoms, MDD appears to be densely connected. This result may be due, in part, to the skip structure that underlies the NSDUH assessment of MDD symptoms. However, it is in line with other results about MDD symptoms in the general population (e.g., Caspi et al., 2014). Second, with 20 out ${7\atopwithdelims ()2} = 21$ possible connections between its seven symptoms, AUD also appears to be densely connected. The estimated associations are less strong than with MDD, which may be due to the skip structure that underlies the assessment of MDD symptoms. Third, there are relatively few estimated connections between the two disorders. Fourth, our edge screening procedure identified a negative association between depressed mood and withdrawal symptoms. Negative associations are scarce in cross-sectional analyses, such as the one reported here.

### Structure Selection

We identified 62 promising edges with our screening procedure, which generates a local median probability structure (LMS, c.f. Fig. 5a). We now wish to find out what the plausible structures are for the data at hand and how the LMS in Fig. 5a relates to the global median probability structure (GMS), i.e., the structure with edges that have marginal posterior inclusion probabilities

$$\begin{aligned} p(\gamma _{ij} = 1 \mid \mathbf {X}) = \sum _{\varvec{\gamma }_s:\gamma _{ij}=1} p(\varvec{\gamma }_s\mid \mathbf {X}) \ge \frac{1}{2}. \end{aligned}$$

Barbieri and Berger (2004) showed that this GMS has, in general, excellent predictive properties. We again use the uniform prior on the structure space, which is consistent with the edge screening results shown above.

We ran the Gibbs sampler for 100, 000 iterations, which visited 62 out of $2^{66}\approx 7e^{19}$ possible structures. Pitting the visited structures against the most frequently visited structure using the Bayes factor,^10^

$$\begin{aligned} \text {BF}_{1s} = \frac{p(\varvec{\gamma }_1 \mid \mathbf {X})}{p(\varvec{\gamma }_s \mid \mathbf {X})} = \frac{p^*( \mathbf {X}\mid \varvec{\gamma }_1)}{p^*( \mathbf {X}\mid \varvec{\gamma }_s)}, \end{aligned}$$

where $\varvec{\gamma }_1$ denotes the most frequently visited model, we identified three structures for which the most visited structure was less than ten times as plausible. A Bayes factor $\text {BF}_{1s}$ of ten or greater is often interpreted to provide strong evidence in favor of $\varvec{\gamma }_1$ (see, for instance Jeffreys, 1961; Lee & Wagenmakers, 2013; Wagenmakers, Love, et al., 2018). The structures for which $\text {BF}_{1s}$ was less than ten, and their estimated posterior structure probabilities, are shown in panels (b), (c) and (d) in Fig. 5. The three structures only differed in the relations between the two disorders.

**Fig. 6:**
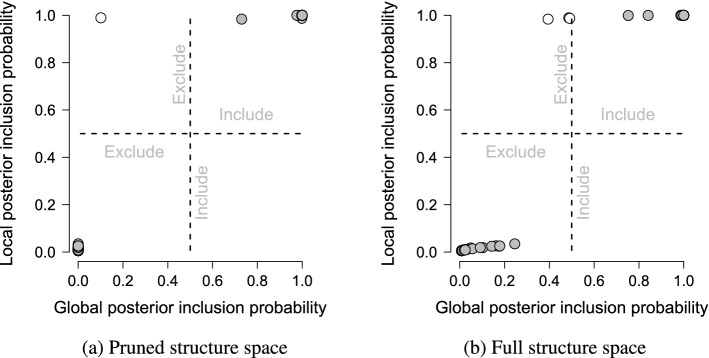
Plots of the local posterior inclusion probabilities of edges against the global posterior inclusion probabilities for the pruned space in (**a**) and the full structure space in (**b**). The dashed lines are the bisection lines.

In Fig. 6a, we plot the posterior inclusion probabilities obtained from the edge screening analysis against those obtained from the structure selection analysis on the pruned structure space. We glean two things from this figure. First, the local inclusion probabilities are at the extremes, i.e., the values zero and one, whereas the global inclusion probabilities show a broader range of values. This difference in separation was also observed in the analysis of simulated data in Fig. 4. The bottom left corner comprises culled edges that have a zero probability of inclusion. Second, there is a great agreement about which edges are or are not in the median probability structure. The LMS and GMS differed in only one edge (indicated in white; points of agreement are in gray). In Fig. 8, we plot the GMS and a difference plot, which reveals the differences between the LMS and GMS (red edges indicating edges that are in the LMS, but not the GMS). Figure 5e shows that the negative association between nodes four and eight is not in the GMS ($p(\gamma _{4,8}=1\mid \mathbf {X}) = .101$). Thus, the LMS produced by our edge screening approach (c.f., Fig. 5a) is an excellent approximation to the GMS identified on the pruned space (c.f., Figs. 5e and 5f). Similar to our simulated example, the edge screening procedure proved to be more liberal than the structure selection approach, i.e., more edges were included in the LMS than in the GMS.

#### Parameter Uncertainty

One of the main benefits of using a Bayesian approach to estimate the network is that it provides a natural framework for quantifying parameter uncertainty. We have two ways to express this uncertainty. The first is the asymptotic posterior distribution based on EM output, which is the posterior distribution associated with the modal structure $\hat{\varvec{\gamma }} = \mathbb {E}(\varvec{\gamma } \mid \hat{\varvec{\Sigma }})$. This is thus a conditional posterior distribution. The second is the model-averaged posterior distribution

$$\begin{aligned}p(\varvec{\Sigma } \mid \mathbf {X}) = \sum _{\varvec{\gamma }} p(\varvec{\Sigma } \mid \mathbf {X}\text {, }\varvec{\gamma }) p(\varvec{\gamma } \mid \mathbf {X})\end{aligned}$$

that can be estimated from the Gibbs sampler’s output.

**Fig. 7:**
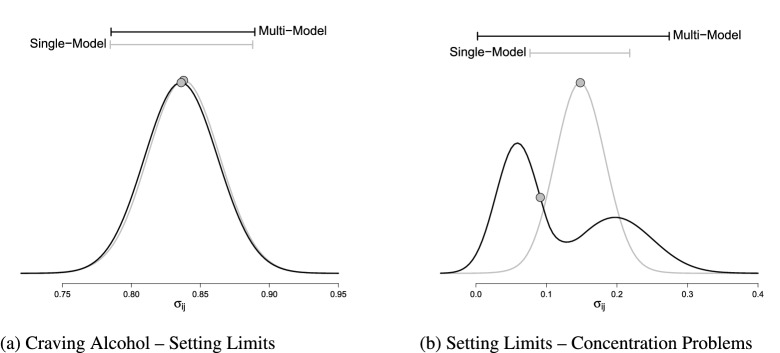
Estimated posterior distributions for two association parameters in the NSDUH example. We plot the asymptotic posterior distributions (i.e., the normal approximations) based on the EM edge screening output in gray, and the model-averaged posterior distribution based on Gibbs sampling in black. The density was estimated using the logspline R package (Kooperberg, 2019). The gray dot reflects the estimated posterior medians. The $95\%$ highest posterior density intervals on top were estimated using the HDInterval R package (Meredith & Kruschke, 2020).

The model-averaged posterior distribution of the network parameters incorporates both the uncertainty that is associated with selecting a structure from the collection of possible structures, and the uncertainty that is associated with the parameters of the individual structures. In this way, the model-averaged posterior distributions offer robust estimates of the network parameters and their uncertainty. Since the model-averaged posterior embraces both sources of uncertainty, the posterior variance of a model-averaged quantity tends to be larger than that of a conditional posterior (i.e., conditioning on a specific structure selected), on average.^11^ For some parameters, this does not lead to striking differences, as Fig. 7a illustrates for one of the associations in the NSDUH data example. In some occasions, however, single-model inference leads us to put faith in a model that assumes parameter values that are not supported by other plausible models. Figure 7b is an illustration of this.

The illustrations above underscore the fact that model-averaging leads to more robust inference on the model parameters than single-model inference (e.g., the output of |rbinnet|’s Edge Screening procedure or the output from |IsingFit|). A benefit of using the Gibbs sampler to estimate the model-averaged posterior distributions is that we can use it’s output to construct model-averaged posterior distributions of other measures of interest. For example, Huth, Luigjes, Marsman, Goudriaan, and van Holst (in press) recently used the Gibbs output to estimate the model-averaged posterior distributions of node centrality measures.

### Structure Selection Without Pruning

To analyze the benefit of our two-step procedure, with edge selection preceding structure selection to prune the structure space, we performed a structure selection analysis without pruning the structure space. We ran the Gibbs sampler for 100, 000 iterations, starting at the posterior mode, which visited 39, 885 out of $2^{120}\approx 1e^{36}$ possible structures. This result immediately underscores the importance of pruning the structure space before structure selection. The posterior structure probabilities of such a large collection of models cannot be estimated with great precision in a reasonable amount of time. Pitting the visited structures against the most frequently visited structure using the Bayes factor identified 52 plausible models. Two questions arise. The first question is about identifying the GMS and how it fares against the LMS identified with edge screening. Second, we wish to determine how the three previously identified structures stack up against the 39, 885 visited structures in the structure selection on the full structure space.

In Fig. 6b, we plot the posterior inclusion probabilities obtained from the edge screening analysis against those obtained from the structure selection analysis on the full structure space. As before, the local inclusion probabilities are mostly located at the extreme ends of zero and one, whereas the global inclusion probabilities are more variable. This difference is emphasized in the bottom left corner of Fig. 6b, since the previously culled edges now received nonzero probabilities. However, Fig. 6b also reveals that there is great agreement about which edges are or are not in the median probability structure. The LMS and GMS on the full structure space differed in three edges.

**Fig. 8:**
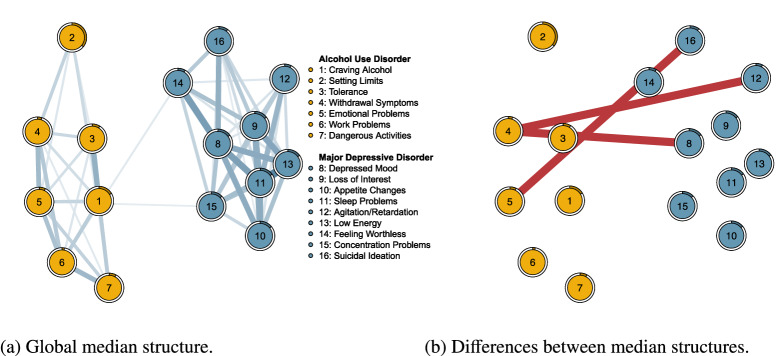
Application of Structure Selection to NSDUH data on the full structure space. **a** indicates the global median probability structure on the full structure space, and **b** indicates the difference between the local and global median probability structures. See text for details. The network plots are produced using the R package qgraph (Epskamp et al., 2012).

In Fig. 8, we plot the GMS from the full space and a difference plot. Figure 8b shows that, as before with the pruned space, the negative association between nodes four and eight is not in the GMS ($p(\gamma _{4,8}=1\mid \mathbf {X}) = .487$). The edge between nodes four and twelve was also not in the second plausible structure observed before (c.f. Fig. 5c; $p(\gamma _{4,12}=1\mid \mathbf {X}) = .394$). The edge between nodes five and sixteen, however, was not screened before ($p(\gamma _{5,16}=1\mid \mathbf {X}) = .491$). In sum, the LMS produced by our edge screening approach (c.f., Fig. 5a) served as a good approximation to the GMS identified on the pruned space (c.f., Figs. 5e and 5f) and on the full space (c.f., Figs. 8a and 8b).

The Gibbs sampler on the full structure space visited 39, 885 structures. Of these 39, 885 structures, 75 were visited between 100 and 1, 450 times, indicating posterior probabilities between .0008 and .015. The remaining 39, 785 structures were visited less than 100 times, indicating a posterior probability of less than .0008. However, in total, the probabilities of these 39, 785 structures added up to .762. Thus, structure selection on the full structure space wastes valuable computational efforts on estimating insignificant structures. This is a prime example of dilution (George, 1999), and once more underscores the importance of pruning the structure space before performing structure selection. The posterior probabilities of the three structures identified earlier were .008, .015 and .008, and with that they were the 7th, 1st and 6th most visited models, respectively. Nevertheless, given the vast amount of visited structures and the tiny probabilities associated with it, their estimates are highly uncertain.

## Discussion

In this paper, we have introduced a novel objective spike-and-slab approach for structure selection for the Ising model, and we have illustrated the full suite of Bayesian tools using simulated and empirical data. The empirical analysis allowed us to underscore the importance of trimming the structure space before its exploration, and that edge screening is capable of identifying relevant edges. The default specification of the spike-and-slab variances resulted in a selection method with consistently high specificity in our simulations, i.e., a low type-1 error rate in edge detection. Posterior estimates of the parameters are easy to obtain for both edge screening and structure selection procedures. Our structure selection procedure opened up the full spectrum of Bayesian tools, and, when paired with edge screening, it quickly zoomed in on plausible structures and promising effects. In sum, we have presented a complete Bayesian methodology for structure determination for the Ising model.

A caveat in our suite of Bayesian tools is the Bayes factor comparing two specific topologies. In principle, we can compute the Bayes factor from the posterior structure probabilities obtained from our structure selection procedure, but only if the Gibbs sampler visited the two structures under scrutiny. However, there is no guarantee that the Gibbs sampler visits the two structures, and even if the Gibbs sampler visits them, their estimated posterior probabilities can be uncertain. We need a more dedicated approach to estimate the Bayes factor if we wish to compare two particular structures of interest. Dedicated procedures have been developed for GGMs (e.g., Williams & Mulder, 2020a; Williams, Rast, Pericchi, & Mulder, 2020) and implemented in the R-package |BGGM| (Williams & Mulder, 2020b). However, these procedures have not yet been developed for the Ising model. We believe that the Laplace approximation that we have used in the paper will be a good starting point for computing the marginal likelihoods. Another option would be the Bridge sampler (Gronau et al., 2017; Meng & Wong, 1996), which fits seamlessly with our Gibbs sampling approach.

In practice, however, we often do not have specific structures that we wish to compare, while we do have hypotheses about entire collections of structures. As an example, consider the hypothesis $\mathcal {H}_1$ that a particular edge should be included in the network. This hypothesis spans the collection of all structures that include the edge. The posterior plausibility for $\mathcal {H}_1$ is therefore the collective plausibility of all structures in the hypothesised collection:

$$\begin{aligned} p(\mathcal {H}_1 \mid \mathbf{X} ) = \sum _{\varvec{\gamma }_s: \gamma _{ij}=1} p(\varvec{\gamma }_s \mid \mathbf{X} ), \end{aligned}$$

i.e., the edge-inclusion probability. The posterior plausibility for the complementary hypothesis $\mathcal {H}_0$ of edge exclusion is $p(\mathcal {H}_0 \mid \mathbf{X} ) = 1- p(\mathcal {H}_1 \mid \mathbf{X} )$. The ratios of the prior and posterior plausibility of these two competing hypotheses then determine the edge-inclusion Bayes factor.^12^ Crucially, the edge-inclusion Bayes factor can quantify the evidence for $\mathcal {H}_1$—edge inclusion—and $\mathcal {H}_0$—edge-exclusion (Jeffreys, 1961; Wagenmakers, Marsman, et al., 2018). Moreover, the Bayes factor can tease apart the *evidence of absence* (i.e., edge-exclusion) from the *absence of evidence*. We therefore believe that the edge-inclusion Bayes factor is a valuable tool for analyzing psychological networks. The methods advocated in this paper—implemented in the R package |rbinnet|—can be used to estimate the edge-inclusion Bayes factors. Huth et al. (in press) recently used it to estimate the evidence for in- and exclusion of edges in networks of alcohol abuse disorder symptoms.

In a recent preprint, Bhattacharyya and Atchade (henceforth BA; 2019) also proposed a continuous spike-and-slab edge selection approach for the Ising model using the pseudolikelihood. The two methods were designed with a different focus, however. Whereas BA focused on networks with many variables, we focused on psychological networks that are relatively small in comparison. As a result, the two approaches differ in several key aspects that make our approach more appealing to analyze psychological networks. For example, BA did not trim the structure space before exploring it with a Gibbs sampler. Our empirical example illustrated why we believe that this is a bad idea. At the same time, we addressed some outstanding issues in this paper that BA left open. For example, BA analyzed the *p* full-conditionals in Eq. (2) in isolation, which provided them an opportunity for fast parallel processing. However, this also forced them to stipulate two independent prior distributions on each focal parameter, which means that they ended up with two posterior distributions for each association. Unfortunately, BA provided no principled solution for combining these estimates for either structure selection or parameter estimation. Another issue is that their spike-and-slab approach required the specification of tuning parameters, but they offered no guidance or automated procedure for their specification. In sum, our method (i) offers an objective specification of the prior distributions that lead to sensible answers, (ii) trims the structure space to circumvent issues related to dilution, and (iii) allows for a meaningful interpretation of the estimated posteriors. Despite these crucial differences, however, the approach of BA is broader than ours, as they also analyzed networks of polytomous variables, while we exclusively focus on the binary case in this paper.

Our specification of the hyperparameters stipulates a mixture of two unit information priors, one fixed and one shrinking, that *a priori* match an approximate credible interval. We chose this setup to mimic the eLasso approach of van Borkulo et al. (2014) and aimed for high specificity. However, researchers might have a different aim and wish to have methods available that have a higher sensitivity (e.g., see the considerations in Epskamp et al., 2017) or that aim for a low false discovery rate instead (e.g., Storey, 2003). In principle, penalty tuning procedures and prior structure probabilities could be tailored to achieve different goals. For example, we could adopt the eLasso approach and select the penalty $\xi$ that minimizes the Bayesian information criterion (BIC; Schwarz, 1978) or the extended BIC (EBIC$_\lambda$, where $\lambda$ is a penalty on complexity; Chen & Chen, 2008) instead of matching the spike-and-slab intersections to credible intervals. These two criteria usually achieve higher sensitivity than Lasso and naturally tie in with the two prior distributions on the structure space that we have used here: A uniform prior distribution on the structure space is consistent with BIC, and a uniform prior distribution on structure complexity is compatible with EBIC$_1$. Furthermore, several alternative prior distributions that account for multiple testing have been discussed in the variable selection literature (e.g., Castillo, Schmidt-Hieber, & van der Vaart, 2015; Womack, Fuentes, & Taylor-Rodriguez, 2015). In sum, there are plenty of options to tailor the spike-and-slab approach to the specific needs of empirical researchers.

The prior specification options that we discussed above are geared towards situations in which researchers have limited or only general ideas about the network they are analyzing. In principle, researchers could have substantive ideas or knowledge about the network under scrutiny, and it is opportune to use this information in its analysis. Prior information could be used to define $\delta$ (e.g., George & McCulloch, 1997) or it could guide the specification of the prior inclusion probabilities of the network’s edges. A parameter’s sign is another common source of information since most relations are usually positive in psychological applications (see Williams & Mulder, 2020a, for an implementation of this idea for GGMs). Investigating how substantive knowledge can be best included in the Bayesian model—and what that implies for the spike-and-slab setup—is another fruitful area of future research.

Implementing our procedures in a compiled language is one of several improvements that we envision for the |rbinnet| package. At this moment, our methods are wholly implemented in R (R Core Team, 2019). Our current implementation of the edge screening procedure implementation is a bit slower than the eLasso implementation in |IsingFit|—the analysis of NSDUH data took approximately 40 seconds for edge screening and 15 seconds for |IsingFit|—structure selection is considerably slower since the Gibbs sampler needs more time to explore the network space. The online appendix contains a simulation to illustrate the running time differences between the different methods and their implementations. There are currently two computational bottlenecks: The specification of the Hessian matrix, and running the Gibbs sampler. Both involve iterating loops that can be computed much faster in a compiled language. Another aspect that we plan to implement shortly is the treatment of missing data. Two options present itself. The first uses selection functions for pairwise removal of missing data points; the second is data-augmentation or imputation. Both methods assume that data are missing at random, or are at least ignorable. The analysis of structurally missing data, e.g., missing data introduced by a skip structure as in our example, requires a different model setup, in principle, and remains an open problem. As for different models, we are currently working on extending the method to Ising models for polytomous (c.f., Bhattacharyya & Atchade, 2019) and ordinal data, and different setups for the spike-and-slab priors. We also plan to implement our software in the open-source statistical software JASP (Love et al., 2019; Wagenmakers, Love, et al., 2018), which would build a user-friendly interface for the functions in |rbinnet|.

### Supplementary Information

Below is the link to the electronic supplementary material.

Supplementary file 1 (pdf 169 KB)

## Appendix A EM Variable Selection for the Pseudolikelihood Ising Model

### The E-Step

The Q-function factors into three distinct terms^13^:

9 $$\begin{aligned} \text {Q}&\left( \left. \varvec{\Sigma }\text {, }\varvec{\mu }\text {, }\theta \,\right| \, \varvec{\Sigma }^k\text {, }\theta ^k\right) \nonumber \\&= \text {Q}_1(\varvec{\Sigma }\text {, }\varvec{\mu } \mid \varvec{\Sigma }^k\text {, }\theta ^k) + \text {Q}_2(\theta \mid \varvec{\Sigma }^k\text {, }\theta ^k) + \text {C}, \end{aligned}$$

where $\text {C} = -\ln (p(\mathbf {X}))$ is a constant term.

The first term in Eq. (9) concerns the pseudoposterior of the Ising model’s parameters

$$\begin{aligned} \text {Q}_1(\varvec{\Sigma }\text {, }\varvec{\mu } \mid \varvec{\Sigma }^k\text {, }\theta ^k) =&\ln \left( p^*(\mathbf {X}\mid \varvec{\mu }\text {, }\varvec{\Sigma })\right) + \ln \left( p(\varvec{\mu })\right) \\&+ \mathbb {E}\left( \left. \ln \left( p(\varvec{\Sigma }\mid \varvec{\gamma })\right) \,\right| \, \varvec{\Sigma }^k\text {, }\theta ^k\right) , \end{aligned}$$

and involves the expectation of the log-transformed spike-and-slab prior

$$\begin{aligned}&\mathbb {E}\left( \left. \ln \left( p(\varvec{\Sigma }\mid \varvec{\gamma })\right) \,\right| \,\varvec{\Sigma }^k\text {, }\theta ^k\right) \\&\quad =\text {C}_1 - \frac{1}{2}\sum _{i=1}^{p-1}\sum _{j=i+1}^p\,\sigma _{ij}^2\,\mathbb {E}\left( \left. \frac{1}{\gamma _{ij}\nu _1 + (1-\gamma _{ij})\nu _0}\,\right| \, \sigma _{ij}^k\text {, }\theta ^k\right) , \end{aligned}$$

where $\text {C}_1$ is a constant term, and the last term can be reformulated as (c.f., Ročková & George, 2014, Eq. 3.6)

$$\begin{aligned} - \frac{1}{2}\sum _{i=1}^{p-1}\sum _{j=i+1}^p\,\sigma _{ij}^2\left\{ \frac{\mathbb {E}\left( \left. \gamma _{ij} \,\right| \, \sigma _{ij}^k\text {, }\theta ^k\right) }{\nu _1}+\frac{1-\mathbb {E}\left( \left. \gamma _{ij} \,\right| \, \sigma _{ij}^k\text {, }\theta ^k\right) }{\nu _0}\right\} , \end{aligned}$$

where the posterior expectation of the selection variable is equal to

10 $$\begin{aligned} \mathbb {E}\left( \left. \gamma _{ij} \,\right| \,\sigma _{ij}^k\text {, }\theta ^k\right) = \frac{p(\sigma _{ij}^k \mid \gamma _{ij} = 1)\, p(\gamma _{ij}=1\mid \theta ^k)}{\sum _{\Gamma _{ij}=\gamma _{ij}}\,p(\sigma _{ij}^k \mid \gamma _{ij})\, p(\gamma _{ij}\mid \theta ^k)}. \end{aligned}$$

The second term in Eq. (9) concerns the posterior distribution of the prior inclusion probability

$$\begin{aligned} \text {Q}_2(\theta \mid \varvec{\Sigma }^k\text {, }\theta ^k)&= \mathbb {E}\left( \left. \ln \left( p(\varvec{\gamma } \mid \theta )\right) \,\right| \,\varvec{\Sigma }^k\text {, }\theta ^k\right) + \ln \left( p(\theta )\right) , \end{aligned}$$

and involves the expectation of the log-transformed prior distribution on the selector variables

$$\begin{aligned}&\mathbb {E}\left( \left. \ln \left( p(\varvec{\gamma } \mid \theta )\right) \,\right| \, \varvec{\Sigma }^k\text {, }\theta ^k\right) \\&\quad =\ln (\theta )\,{p\atopwithdelims ()2}+\ln \left( \frac{\theta }{1-\theta }\right) \,\sum _{i=1}^{p-1}\sum _{j=i+1}^p\, \mathbb {E}\left( \left. \gamma _{ij} \,\right| \, \sigma _{ij}^k\text {, }\theta ^k\right) , \end{aligned}$$

and is also readily computed using the expression in Eq. (10).

#### The M-Step

We separately optimize the two components of the Q-function in the M-step. Unfortunately, there is no closed-form solution for the maximization of $\text {Q}_1$, and we approximate the M-Step using a single iteration of a Newton-Raphson algorithm (Lange, 1995; Tanner, 1996). The details are in “Appendix B”. The maximization of $\text {Q}_2$ is in closed-form,

$$\begin{aligned} \theta ^{k+1} = \frac{\sum _{i=1}^{p-1}\sum _{j=i+1}^p \mathbb {E}\left( \gamma _{ij}\mid \sigma _{ij}^{k}\text {, }\theta ^{(k)}\right) + \alpha - 1}{\alpha + \beta + {p \atopwithdelims ()2} - 2}. \end{aligned}$$

#### Posterior Standard Deviations

The EM algorithm provides us with an estimate of a local posterior mode, and we seek a way to quantify the uncertainty in this modal estimate. We express this uncertainty using the variance-covariance matrix of the normal approximation to the posterior (Tanner, 1996, e.g.,), i.e., the inverse of the Hessian matrix. The Hessian matrix is computed in the M-step of our EMVS approach, see “Appendix B”, and serves as an estimate of the variance-covariance matrix of the complete posterior $p(\varvec{\Sigma }\text {, }\varvec{\mu }\text {, }\theta \text {, }\varvec{\gamma } \mid \mathbf {X})$. To estimate the variance-covariance matrix of the marginal posterior $p(\varvec{\Sigma }\text {, }\varvec{\mu }\text {, }\theta \mid \mathbf {X})$, we have to use the inverse of the Hessian subject to the marginal spike-and-slab prior distributions on the interaction effects,

$$\begin{aligned} p(\sigma _{ij} \mid \theta ) = \theta \, \frac{1}{\sqrt{2\pi \nu _1}}\,e^{\left( -\frac{1}{2\nu _1}\sigma _{ij}^2\right) } + (1-\theta ) \, \frac{1}{\sqrt{2\pi \nu _0}}\,e^{\left( -\frac{1}{2\nu _0}\sigma _{ij}^2\right) }. \end{aligned}$$

For prior specification—setting the values of $\nu _1$—we use the inverse Hessian excluding prior distributions on the parameters.

## Appendix B The M-Step approximation for $\text {Q}_1$

We approximate the M-step of $\text {Q}_1$ using a single iteration of the Newton–Raphson algorithm. Let $\varvec{\eta } = \{\mu _1\text {, }\dots \text {, }\mu _p\text {, }\sigma _{12}\text {, }\dots \text {, }\sigma _{(p-1)p}\}$ denote the $\left( {p\atopwithdelims ()2} + p\right) \times 1$ vector of pseudolikelihood parameters. Then, the Newton–Raphson iteration is equal to

$$\begin{aligned} \varvec{\eta }^{k+1} = \varvec{\eta }^k + \mathbf{H} ^{-1}{} \mathbf{D} , \end{aligned}$$

where $\mathbf{D}$ is the $\left( {p\atopwithdelims ()2} + p\right) \times 1$ vector of first-order partial derivatives and $\mathbf{H}$ the $\left( {p\atopwithdelims ()2} + p\right) \times \left( {p\atopwithdelims ()2} + p\right)$ matrix of second-order partial derivatives, i.e., the Hessian matrix.

The first-order partial derivatives are equal to

$$\begin{aligned} \frac{\partial }{\partial \mu _i} \text {Q}_1 = \sum _{v = 1}^n x_{vi} - \sum _{v = 1}^n \frac{\exp \left( \mu _{i} + \sum _{j \ne i}\sigma _{ij}x_{vj}\right) }{1+\exp \left( \mu _{i} + \sum _{j \ne i}\sigma _{ij}x_{vj}\right) } - \mu _i \end{aligned}$$

for the main effects, and

$$\begin{aligned} \frac{\partial }{\partial \sigma _{ij}} \text {Q}_1&= 2\sum _{v=1}^n x_{vi}x_{vj}\\&- \sum _{v = 1}^n x_{vj}\frac{\exp \left( \mu _{i} + \sum _{q \ne i}\sigma _{iq}x_{vq}\right) }{1+\exp \left( \mu _{i} + \sum _{q \ne i}\sigma _{iq}x_{vq}\right) }\\&- \sum _{v = 1}^n x_{vi}\frac{\exp \left( \mu _j + \sum _{q \ne j}\sigma _{jq}x_{vq}\right) }{1+\exp \left( \mu _{j} + \sum _{q \ne j}\sigma _{jq}x_{vq}\right) }\\&- \sigma _{ij} \left\{ \frac{\mathbb {E}(\gamma _{ij} \mid \sigma _{ij}^k\text {, }\theta ^k)}{\nu _1}+\frac{1-\mathbb {E}(\gamma _{ij} \mid \sigma _{ij}^k\text {, }\theta ^k)}{\nu _0}\right\} \\&= 2\sum _{v=1}^n x_{vi}x_{vj}- \sum _{v = 1}^n x_{vj}\,p^*_{vi} - \sum _{v = 1}^n x_{vi}\,p^*_{vj}- \sigma _{ij} e_{ij} \end{aligned}$$

for the interaction effects. Here, we have used $p^*_{vi}$ to denote the conditional probability $p(X_i = 1 \mid \mathbf {x}_{v}^{(i)})$ and $e_{ij}$ to denote the expected precision.

The Hessian matrix is slightly more complicated, as it requires some tedious bookkeeping. To emphasize its structure and ease its derivation, we split the Hessian matrix in four components,

$$\begin{aligned} \mathbf {H} = \left( \begin{array}{ll} \mathbf {H}_\mu &{} \mathbf {H}_{\mu \,\Sigma }\\ \mathbf {H}_{\mu \,\Sigma }^\mathsf{T} &{} \mathbf {H}_{\Sigma } \end{array}\right) , \end{aligned}$$

where $\mathbf{H} _\mu$, and $\mathbf{H} _\Sigma$ are the second-order partial derivatives of the main effects $\varvec{\mu }$ and $\varvec{\Sigma }$, respectively, and $\mathbf{H} _{\mu \,\Sigma }$ their cross-derivatives. The submatrix $\mathbf{H} _\mu$ is diagonal and has elements

$$\begin{aligned} \frac{\partial ^2}{\partial \mu _i \partial \mu _j} \text {Q}_1 = {\left\{ \begin{array}{ll} - \sum _{v = 1}^n p^*_{vi}(1-p^*_{vi}) - 1&{} \text { if } i = j\\ 0 &{} \text { if } i \ne j \end{array}\right. } \end{aligned}$$

The submatrix $\mathbf{H} _{\mu \, \Sigma }$ has elements

$$\begin{aligned} \frac{\partial ^2}{\partial \mu _i \partial \sigma _{rj}} \text {Q}_1 = {\left\{ \begin{array}{ll} - \sum _{v = 1}^n x_{vj}\, p^*_{vi}(1-p^*_{vi})&{} \text { if } r = i\\ 0 &{} \text { if } i \ne r \end{array}\right. } \end{aligned}$$

Finally, the submatrix $\mathbf{H} _{\Sigma }$ has diagonal elements

$$\begin{aligned} \frac{\partial ^2}{\partial \sigma _{ij}^2} \text {Q}_1 = - \sum _{v = 1}^n x_{vj}\,p^*_{vi}q^*_{vi} - \sum _{v = 1}^n x_{vi}\,p^*_{vj}q^*_{vj}- e_{ij}, \end{aligned}$$

and off-diagonal elements

$$\begin{aligned} \frac{\partial ^2}{\partial \sigma _{ij} \partial \sigma _{ru}} \text {Q}_1 = {\left\{ \begin{array}{ll} - \sum _{v = 1}^n x_{vj}x_{vu}\,p^*_{vi}q^*_{vi} &{}\text { if } i = r \text { and } j \ne u\\ - \sum _{v = 1}^n x_{vj}x_{vr}\,p^*_{vi}q^*_{vi} &{}\text { if } i = u \text { and } j \ne r\\ - \sum _{v = 1}^n x_{vi}x_{vr}\,p^*_{vj}q^*_{vj} &{}\text { if } i \ne r \text { and } j = u\\ - \sum _{v = 1}^n x_{vi}x_{vu}\,p^*_{vj}q^*_{vj} &{}\text { if } i \ne u \text { and } j = r\\ 0 &{}\text { if } i \ne u \text { and } j \ne r \end{array}\right. } \end{aligned}$$

where $$q_{vj}^*= 1 - p_{vj}^*$$.

## Appendix C A Gibbs Sampling Routine for Structure Selection

The Gibbs sampler iterates between the following five steps. If a uniform prior is stipulated on the structure space, then step four is skipped.

*Step 1. Sampling the main effects *$\mu _i$. With the assumption of prior independence, the main effects are also found to be independent *a posteriori* and do not depend on the selection variables $\varvec{\gamma }$. Given the standard normal prior distribution, the full-conditional posterior distribution $p(\mu _i \mid \varvec{\sigma }_i \text {, } \varvec{\omega }_i \text {, }\mathbf {X})$ of the main effect $\mu _i$ is a normal distribution, with mean

$$\begin{aligned} \frac{x_{i+} - \frac{n}{2} - \sum _{v=1}^n\sum _{j\ne i}\sigma _{ij}x_{jv}\omega _{iv}}{1+\omega _{i+}} \end{aligned}$$

and variance $(1 + \omega _{i+})^{-1}$, where we have used $\varvec{\sigma }$ to denote the $p - 1 \times 1$ vector

$$\begin{aligned} \varvec{\sigma } = (\sigma _{i1}\text {, }\dots \text {, }\sigma _{i(i-1))}\text {, }\sigma _{i(i+1)}\text {, }\dots \text {, }\sigma _{ip})^\mathsf{T}, \end{aligned}$$

and $x_{i+}$ and $\omega _{+i}$ to denote the margins $\sum _{v=1}^nx_{iv}$ and $\sum _{v=1}^n\omega _{vi}$, respectively.

*Step 2. Sampling the interaction effects* $\sigma _{ij}$. Given $\gamma _{ij}$, the prior distribution for $\sigma _{ij}$ is normal with a zero mean, and variance $\phi = \gamma _{ij}\nu _{1} + (1-\gamma _{ij})\nu _0$. The full-conditional posterior distribution is then a normal distribution with mean

$$\begin{aligned}&\left( \varvec{\omega }_i^\mathsf{T}\mathbf {x}_j + \varvec{\omega }_j^\mathsf{T}\mathbf {x}_i + \phi ^{-1}\right) ^{-1}\,\times \, \left( 2\mathbf {x}_i^\mathsf{T}\mathbf {x}_j -\frac{1}{2}x_{+i}-\frac{1}{2}x_{+j} \right. \\&\left. - \sum _{v=1}^n\omega _{vi}x_{vj}\left[ \mu _i + \sum _{q \ne i \ne j}\sigma _{iq}x_{vq}\right] - \sum _{v=1}^n\omega _{vj}x_{vi}\left[ \mu _{j} + \sum _{q\ne i\ne j}\sigma _{jq}x_{vq} \right] \right) \end{aligned}$$

and variance $\left( \varvec{\omega }_i^\mathsf{T}\mathbf {x}_j + \varvec{\omega }_j^\mathsf{T}\mathbf {x}_i + \phi ^{-1}\right) ^{-1}$, where we have used $\mathbf {x}_i$ to denote the $n \times 1$ vector with elements $[x_{iv}]$.

*Step 3. Sampling the inclusion variables* $\gamma _{ij}$. The full-conditional posterior distribution of $\gamma _{ij}$ is a Bernoulli distribution with probability of inclusion:

$$\begin{aligned} p(\gamma _{ij} = 1 \mid \sigma _{ij}\text {, }\theta ) = \frac{1}{1 + \frac{1-\theta }{\theta } \,\exp \left( \frac{1}{2(\nu _1-\nu _0)}\sigma _{ij}^2\right) }. \end{aligned}$$

*Step 4. Sampling the prior inclusion probability* $\theta$. The full-conditional posterior distribution of $\theta$ is a Beta distribution, with parameters

$$\begin{aligned} \alpha&= 1 + \gamma _{++} / 2,\\ \beta&= 1 + {{p}\atopwithdelims (){2}} - \gamma _{++} / 2, \end{aligned}$$

where $$\gamma _{++} = \sum _{i=1}^p\sum _{j=1}^p\gamma _{ij}$$.

*Step 5. Sampling the augmented variables* $\omega _{vi}$. The full-conditional posterior distribution of $\omega _{vi}$ is proportional to

$$\begin{aligned} p\left( \omega _{vi} \mid \varvec{\sigma }_i\text {, }\mathbf {x}_v^{(i)}\right) \propto \exp \left( -\frac{1}{2}\omega _{vi}\left( \mu _i + \sum _{j \ne i}\sigma _{ij}x_{vj}\right) ^2\right) \,p(\omega _{vi}), \end{aligned}$$

where $p(\omega _{vi}) = p(\omega _{vi} \mid 1\text {, }0)$ is a Pólya-Gamma distribution with parameters $b = 1$ and $c = 0$. Polson et al. (2013a) show that the Pólya-Gamma distribution with parameters $b = 1$ and $c \ne 1$ is equal to an exponential tilting of the Pólya-Gamma distribution with parameters $b = 1$ and $c = 0$,

$$\begin{aligned} p(\omega \mid 1\text {, }c) = \frac{\exp \left( -\frac{1}{2}c^2\omega \right) \, p(\omega \mid 1\text {, }0)}{\cosh \left( \frac{1}{2}c\right) }, \end{aligned}$$

which consequently shows that $p\left( \omega _{vi} \mid \varvec{\sigma }_i\text {, }\mathbf {x}_v^{(j)}\right)$ is a Pólya-Gamma distribution with parameters $b = 1$ and $c = \mu _i + \sum _{j \ne i}\sigma _{ij}x_{vj}$. These values can be simulated using the R (R Core Team, 2019) programs BayesLogit (Polson, Scott, & Windle, 2013b) and BayesReg (Makalic & Schmidt, 2016).

## Footnotes

1 While our manuscript was under review, Ly and Wagenmakers (2021) elegantly extended this inconsistency result to any statistical model using techniques similar to ours.

2 The package can be downloaded from https://github.com/MaartenMarsman/rbinnet/.

3 The analysis of structure selection consistency that we discuss here applies to either the edge screening procedure or the structure selection procedure that we present in the next sections. It does not, however, pertain to the situation of structure selection after initial edges have been screened.

4 This $\mathcal {S}$-closed view might be unrealistic in practice. If the correct structure is not in $\mathcal {S}$, *t* is the structure closest in Kullback–Leibler divergence to the true structure.

5 We estimate $\text {Var}(\hat{\sigma }_{ij})$ by setting it to the diagonal of the Fisher information matrix for the pseudolikelihood, evaluated at the maximum pseudolikelihood estimate, c.f., “Appendix 7”. Alternatively, one could approximate the variance by running a Gibbs sampling approach using non-informative priors. This, however, would take considerably more time in practice.

6 With the caveat that Barbieri and Berger (2004) define the posterior inclusion probability of an edge *i*–*j* to be the aggregate of the posterior probabilities of structures that include the edge

$$\begin{aligned} p(\gamma _{ij} = 1 \mid \mathbf {X}) = \sum _{\varvec{\gamma }_s: \gamma _{ij} = 1} p(\varvec{\gamma }_s \mid \mathbf {X}) \end{aligned}$$

where we define the posterior inclusion probabilities locally.

7 If we use $\mathcal {S}^*$ to denote the pruned structure space, then $\mathcal {S}^*\subset \mathcal {S}$. This implies that the absolute magnitude of the posterior structure probability will be higher when calculated over the pruned space:

$$\begin{aligned} \frac{p(\mathbf {X} \mid \varvec{\gamma }) \,p(\varvec{\gamma })}{\sum _{\varvec{\gamma } \in \mathcal {S}^*} p(\mathbf {X} \mid \varvec{\gamma }) \,p(\varvec{\gamma })} \ge \frac{p(\mathbf {X} \mid \varvec{\gamma }) \,p(\varvec{\gamma })}{\sum _{\varvec{\gamma } \in \mathcal {S}} p(\mathbf {X} \mid \varvec{\gamma }) \,p(\varvec{\gamma })}, \end{aligned}$$

since there are less terms in the denominator of the ratio on the left. But, for any two structures $\varvec{\gamma }_i$ and $\varvec{\gamma }_j$ in the pruned space $\mathcal {S}^*$, the posterior odds are

$$\begin{aligned} \frac{p(\varvec{\gamma }_i \mid \mathbf {X})}{p(\varvec{\gamma }_i \mid \mathbf {X})} = \frac{p(\mathbf {X} \mid \varvec{\gamma }_i) p(\varvec{\gamma }_i)}{p(\mathbf {X} \mid \varvec{\gamma }_j) p(\varvec{\gamma }_j)}, \end{aligned}$$

in which the denominator does not play a role. Thus, even though the absolute values of the posterior probabilities for specific structures differ between the pruned and the full structure space, their relative probabilities do not. The upside is that after pruning the edge set, the remaining model probabilities are estimated with increased precision. This result does assume that the prior on the topologies is unaffected by pruning, i.e., it is formulated on the full structure space $\mathcal {S}$.

8 We follow the simulation setup of van Borkulo et al. (2014) and set the association parameters $\sigma _{ij}$ equal to $|Z_{ij}|$, where the $Z_{ij}$ are independent normal random variables with mean zero and variance 0.25. The main effect parameters $\mu _i$ are set to $-|Z_i|$, where $Z_i$ is an independent normal random variable with mean $\sigma _{i+}=\sum _{j = 1}^p\sigma _{ij} / 2$, with $\sigma _{ii}=0$, and variance $\sigma _{i+}^2/36$.

9 In the sparse setting, one expects to detect approximately $\sqrt{\tfrac{n}{log(p (p-1)/2)}}$ edges with Lasso given a penalty of approximately $\sqrt{\tfrac{log(p (p-1)/2)}{n}}$. This translates to approximately 20 edges with 2, 000 observations and 60 with 20, 000 observations. In this simulation, we used a dense structure setup, a setup for which eLasso was not designed. The penalty values selected by eLasso could be used to analyze the performance of eLasso in practical situations, where the lower bound would be approximately $\sqrt{\tfrac{log(p (p-1)/2)}{n}}$. Here, the selected penalty values ranged between 0.0153 and 0.0319 with 2, 000 observations. These values range below the lower bound of 0.0482. With 20, 000 observations, we find values ranging between 0.0057 and 0.0158, which also largely range below the lower bound of 0.0153. This indicates that eLasso has difficulty with the non-sparse setting simulated here.

10 For the hierarchical setup, we have to include the prior structure probabilities in this equation:

$$\begin{aligned} \text {BF}_{1s} = \frac{p(\varvec{\gamma }_1 \mid \mathbf {X})}{p(\varvec{\gamma }_s \mid \mathbf {X})}\times \frac{p(\varvec{\gamma }_s)}{p(\varvec{\gamma }_1)} = \frac{p^*( \mathbf {X}\mid \varvec{\gamma }_1)}{p^*( \mathbf {X}\mid \varvec{\gamma }_s)} \end{aligned}$$

where $p(\varvec{\gamma }_s)$ is the beta-binomial distribution

$$\begin{aligned} {{p \atopwithdelims ()2} \atopwithdelims ()\gamma _{s\text {, }++}} \frac{\text {B}\left( \gamma _{s\text {, }++} + \alpha , \text {, } {p\atopwithdelims ()2} - \gamma _{s\text {, }++} + \beta \right) }{\text {B}\left( \alpha , \text {, } \beta \right) },\end{aligned}$$

with $$\alpha = \beta = 1$$.

11 From the law of total variance: $\text {Var}(\Sigma \mid \mathbf {X}) \ge \mathbb {E}(\text {Var}(\Sigma \mid \mathbf {X}\text {, }\varvec{\Gamma }))$.

12 The structure selection approach advocated in this paper offers unbiased estimates of these posterior probabilities and can be used to compute the edge-inclusion Bayes factor. However, as mentioned in Footnote 7, the edge screening step might inflate some of the involved probabilities, so we advocate not to use edge screening when the aim is to compute the posterior plausibility of $\mathcal {H}_1$ and $\mathcal {H}_0$. More research is needed to investigate the impact of edge screening on the edge-inclusion Bayes factor.
