# Supplementary material for: Objective Bayesian Edge Screening and Structure Selection for Ising Networks
Source: Psychometrika. 2022 Feb 22;87(1):47–82. doi: 10.1007/s11336-022-09848-8 (PMC9021150; doi:10.1007/s11336-022-09848-8)
Supplement: Supplementary file 1 — (pdf 169 KB) [file 11336_2022_9848_MOESM1_ESM.pdf]

Online Appendix – Objective Bayesian Edge Screening and Structure Selection for Ising Networks

***
***

This is the online appendix for the paper "Objective Bayesian Edge Screening and Structure Selection for Ising Networks."

A Comparison of Bayesian Approaches to Structure Selection for Networks of Binary Data

We have introduced novel edge screening and structure selection procedures for estimating the topology of Ising networks. As discussed in our paper, other Bayesian approaches exist for structure selection for the Ising model (Pensar et al., 2017)—implemented in the R-package `BDgraph` (Mohammadi and Wit, 2019)—and the multivariate probit model (Talhouk et al., 2012)—implemented in the R-package `BGGM` (Williams and Mulder, 2020b). We wish to compare the performance of these two approaches to that of our procedures in terms of edge-selection sensitivity and specificity.

Similar to the other simulations in our paper, we focus on simulations in which and Erdős and Rényi (1960) model is used to generate the topologies. The parameters corresponding to present edges are simulated uniformly between 0.5 and 2, and set to zero otherwise. In the simulations, we vary π, the probability of generating an edge between two variables, p, the number of variables, and n, the number of observations and generate 100 datasets for each combination of values for π, p, and n.

We analyze the simulated datasets using the default settings of `rbinnet` (`precision` = .975), `BDgraph`, and `BGGM`, except that for the latter we varied the specification of the `delta` parameter. We have used `delta` values of 5, and 24, which translate into a prior standard deviation for the partial correlation between the latent Gaussian variables of approximately .41, and .2, respectively. These `delta` values were suggested in Williams and Mulder (2020a). We ran each of the sampling-based methods —structure selection, `BDgraph` and `BGGM`—for $10,000$ iterations. Sensitivity and Specificity were computed as defined in our paper. Table 1 shows the results for a generating probability $\pi = 0.1$ and Table 2 shows the results for $\pi = 0.5$.

With increasing sample size, the sensitivity increases for all methods. In Table 1, sensitivity starts around 13% for most methods (36% for `BGGM`, `delta` $= 24$), it increases to around 80% (86% for `rbinnet`, edge screening). In Table 2, which considers denser networks, sensitivity starts around 85% and increases to around 100%. The same observation could be made for specificity. In Table 1, specificity starts around 95% for most methods (87% for `BGGM`, `delta` $= 24$), it increases to 95% and above for all methods. In Table 2, specificity already starts above 95% and slowly increases to over 99% for all methods, except that

		n			
		250	500	$1,000$	10,000
Screening	SEN	.133	.193	.438	.859
	SPE	.954	.959	.966	.953
Selection	SEN	.141	.167	.394	.807
	SPE	.941	.964	.978	.987
Screening & Selection	SEN	.133	.172	.394	.807
	SPE	.954	.963	.975	.987
`BDgraph`	SEN	.117	.151	.306	.808
	SPE	.949	.976	.988	.988
`BGGM` (`delta` = 5)	SEN	.133	.163	.317	.802
	SPE	.941	.972	.985	.995
`BGGM` (`delta` = 24)	SEN	.362	.297	.446	.802
	SPE	.874	.921	.959	.996

Table 1
Sensitivity and specificity for $\pi = 0.1$, as a measure of performance of the methods implemented in `rbinnet`, BDgraph, and BGGM.

for edge screening it appears to hover around 97%. There are no major differences in the performance of the different methods, except that or smaller sample sizes `BGGM`—using a `delta` value equal to 24—showed an increased sensitivity and reduced specificity.

Computing Time Differences Between the Methods and Their Implementations

The methods are currently implemented in the `rbinnet` package using native R code. We therefore expect it to run considerably slower than other packages, such as `BGGM` and `BDgraph`, which run code in a compiled language. To get a sense of the differences in running time between the different methods and implementation, we simulated data for $n = 200$, 500, and $1,000$ cases for a $p = 10$, and 15 variable network and ran the different procedures. The samplers each ran for $10,000$ iterations. Simulations ran on a MacBook pro with an Intel i5 CPU and clock speed 2.0 Ghz and 16Gb of memory.

The results are shown in Table 3. Observe that edge screening and eLasso are both really fast. But these only do optimization. When comparing the simulation based methods, we observe that structure selection is much slower than both `BGGM` and `BDgraph`. Whereas `BGGM` is roughly four to six times faster than structure selection for the $p = 10$ variable network, it is seven to eight times faster for the $p = 15$ variable network. Thus, `BGGM` becomes relatively faster than structure selection for increasing network sizes. Interestingly, the relative speed difference shrinks with increasing sample sizes. `BDgraph`, on the other hand, is roughly 30 to 50 times faster than structure selection. In contrast to the implementation of `BGGM`, `BDgraph` does not become relatively faster than structure selection when the network's size increases, although it does appear to become relatively faster for increasing sample sizes.

		n			
		250	500	$1,000$	10,000
Screening	SEN	.846	.966	.994	1.000
	SPE	.978	.959	.971	.964
Selection	SEN	.859	.966	.991	1.000
	SPE	.970	.957	.983	.995
Screening & Selection	SEN	.846	.960	.994	1.000
	SPE	.978	.962	.983	.995
`BDgraph`	SEN	.815	.895	.948	1.000
	SPE	.977	.992	.995	.999
`BGGM` (`delta` = 5)	SEN	.816	.934	.977	1.000
	SPE	.984	.994	.989	.992
`BGGM` (`delta` = 24)	SEN	.882	.957	.986	1.000
	SPE	.956	.988	.975	.989

Table 2

Sensitivity and specificity for $\pi = 0.5$, as a measure of performance of the methods implemented in `rbinnet`*, BDgraph, and BGGM.*

p	n	Screening	Selection	`BGGM`	`BDgraph`	`IsingFit`
10	200	0.4	55.4	10.1	1.4	0.3
	500	0.5	84.1	19.7	2.0	0.3
	1,000	0.7	166.4	38.9	4.9	0.4
15	200	1.1	150.8	19.0	4.7	0.5
	500	1.3	342.2	44.7	7.7	0.6
	1,000	2.2	640.0	88.9	13.5	0.8

Table 3

Running time in seconds for the different methods and implementations in `rbinnet`*,* `BDgraph`*, BGGM, and* `IsingFit`*. The simulation based methods ran for $10,000$ iterations each.*

References

Erdős, P. and Rényi, A. (1960). On the evolution of random graphs. *Publications of the Mathematical Institute of the Hungarian Academy of Sciences*, 5(1):17–60.

Mohammadi, R. and Wit, E. (2019). BDgraph: An R package for Bayesian structure learning in graphical models. *Journal of Statistical Software*, 89(3).

Pensar, J., Nyman, H., Niiranen, J., and Corander, J. (2017). Marginal pseudo-likelihood learning of discrete Markov network structures. *Bayesian Analysis*, 12(4):1195–1215.

Talhouk, A., Doucet, A., and Murphy, K. (2012). Efficient Bayesian inference for multivariate probit models with sparse inverse covariance matrices. *Journal of Computational and Graphical Statistics*, 21(3):739–757.

Williams, D. R. and Mulder, J. (2020a). Bayesian hypothesis testing for Gaussian graph-

ical models: Conditional independence and order constraints. *Journal of Mathematical Psychology*, 99(102441).

Williams, D. R. and Mulder, J. (2020b). BGGM: Bayesian Gaussian graphical models in R. *Journal of Open Source Software*, 5(51):2111.
